# Dihydromyricetin Promotes Glucagon‐Like Peptide‐1 Secretion and Improves Insulin Resistance by Modulation of the Gut Microbiota‐CDCA Pathway

**DOI:** 10.1002/mnfr.202400491

**Published:** 2025-03-13

**Authors:** Pengfei Li, Yong Zhang, Hedong Lang, Pengfei Hou, Yu Yao, Ruiliang Zhang, Xiaolan Wang, Qianyong Zhang, Mantian Mi, Long Yi

**Affiliations:** ^1^ Research Center for Nutrition and Food Safety, Chongqing Key Laboratory of Nutrition and Health, Chongqing Medical Nutrition Research Center Institute of Military Preventive Medicine, Third Military Medical University Chongqing P.R. China

**Keywords:** chenodeoxycholic acid, dihydromyricetin, glucagon‐like peptide‐1, gut microbiota, insulin resistance

## Abstract

Insulin resistance is a common metabolic disease, and its pathogenesis is still unclear. The decrease of glucagon‐like peptide‐1 (GLP‐1) level mediated by the alteration of gut microbiota may be the pathogenesis. The study was to investigate the regulatory effect of dihydromyricetin (DHM) on GLP‐1 level and insulin resistance induced by high‐fat diet (HFD), and to further explore its possible molecular mechanism. Mice were fed an HFD to establish the model of insulin resistance to determine whether DHM had a protective effect. DHM could improve insulin resistance. DHM increased serum GLP‐1 by improving intestinal GLP‐1 secretion and inhibiting GLP‐1 decomposition, associated with the alteration of intestinal intraepithelial lymphocytes (IELs) proportions and decreased expression of CD26 in IELs and TCRαβ^+^ CD8αβ^+^ IELs in HFD‐induced mice. DHM could ameliorate GLP‐1 level and insulin resistance by modulation of gut microbiota and the metabolites, particularly the regulation of chenodeoxycholic acid (CDCA) content, followed by the inhibition of farnesoid X receptor (FXR) expression in intestinal L cells and increased glucagon gene (Gcg) mRNA expression and GLP‐1 secretion. This research demonstrates the role of “gut microbiota‐CDCA” pathway in the improvement of intestinal GLP‐1 levels in HFD‐induced mice by DHM administration, providing a new target for the prevention of insulin resistance.

## Graphical abstract

Dihydromyricetin (DHM) administration improved insulin resistance in HFD‐induced mice through the increased serum GLP‐1 level. This benefits was mediated by the decreased degradation of GLP‐1 associated with the reduced proportion of TCRαβ^+^ CD8αβ^+^ IELs and decreased expression of CD26/DPP‐4 in IELs, as well as the increased GLP‐1 secretion by intestinal L cells through the “gut microbiota‐CDCA” pathway.

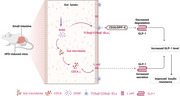

**Abbreviations**
Abx: antibiotic
ATCC: American Type Culture Collection
AUC: area under the curve
BSH: bile salt hydrolase
CA: cholic acid
CCK‐8: cell counting kit‐8
CDCA: chenodeoxycholic acid
CHO: cholesterol
DHM: dihydromyricetin
DPP‐4: dipeptidyl peptidase‐4
ELISA: enzyme linked immunosorbent assay
FXR: farnesoid X receptor
Gcg: glucagon gene
GLP‐1: glucagon‐like peptide‐1
HDL‐C
: high‐density lipoprotein cholesterol
HFD: high‐fat diet
IEC: intestinal epithelial cell
IEL: intestinal intraepithelial lymphocyte
IPGTT: intraperitoneal glucose tolerance test
ITT: insulin tolerance test
LDL‐C: low‐density lipoprotein cholesterol
OGTT: oral glucose tolerance test
OTU: operational taxonomic units
PBS: phosphate buffered saline
PCoA: principal Coordinates Analysis
QC: quality control
RT‐qPCR: real time quantitative polymerase chain reaction
SPF: specific pathogen free
T2DM: diabetes mellitus Type 2
TCA
: taurocholate acid
TG: triacylglycerol
TUDCA: tauroursodeoxycholic acid
T‐β‐MCA: tauro‐β‐muricholic acid
Z‐Gug: Z‐guggulsterone

## 1 Introduction

Insulin resistance was defined as the inability of insulin to optimally stimulate glucose transport to body cells (hyperinsulinemia or impaired glucose tolerance) [1]. Over the past decades, it has become clear that insulin resistance is a common risk factor for many diseases, including diabetes mellitus Type 2 (T2DM), cardiovascular diseases, and endocrine diseases [2, 3]. There are currently no drugs specifically approved for the treatment of insulin resistance, but various studies have demonstrated the efficacy of certain antidiabetic drugs in improving insulin resistance [1], such as glucagon‐like peptide‐1 (GLP‐1) receptor agonists (GLP‐1RA) [4]. GLP‐1 is an incretin secreted by L cells of intestinal mucosa [5]. It has the function of enhancing GLu‐dependent insulin secretion, inhibiting glucagon secretion, slowing gastric emptying to reduce blood GLu, and alleviating insulin resistance [6, 7]. GLP‐1 is rapidly degraded by intestinal CD26/DPP‐4 (dipeptidyl peptidase IV) after production [6]. DPP‐4/CD26, a serine protease belonging to the Type II transmembrane glycoprotein family, is expressed on the surface of T cells, B cells, and myeloid cells [8]. Studies show that serum GLP‐1 level is significantly lower in patients with insulin resistance, so maintaining the level of GLP‐1 is of great significance to prevent or delay the development of insulin resistance [5]. Although GLP‐1RAs have achieved some efficacy in the treatment of insulin resistance, there are also some drawbacks, such as side effects, poor compliance, and drug resistance [9, 10]. Thereby, approaches aimed at boosting the endogenous production or release of GLP‐1 is regarded as an innovative approach for treating insulin resistance.

Dihydromyricetin (DHM) is a polyphenolic phytochemical found in plants such as *Ampelopsis grossedentata* [11]. Our previous studies found that regular DHM supplementation could significantly alleviate the metabolic abnormalities of fasting blood glucose, glycosylated hemoglobin, and cystatin C in adults with Type 2 diabetes [12]. DHM notably promoted the maintenance of intestinal mucosal barrier integrity through regulating gut microbiota and short‐chain fatty acids in nonalcoholic steatohepatitis (NASH) C57BL/6 mice model induced by high‐fat diet (HFD). In addition, DHM significantly reversed the elevated blood glucose abnormally in HFD‐induced mice [13]. DHM could also increase serum GLP‐1 level and enhance exercise endurance in aerobic exercise mice [14], indicating that DHM may play a role in preventing and treating chronic diseases by regulating gut microbiota or its metabolites to increase GLP‐1 level in vivo. Published literatures indicated that DHM combined with *L*acticaseibacillus *rhamnosus* 1.0320 restored the intestinal barrier induced by acute alcohol exposure by upregulating the intestinal short‐chain fatty acid levels [15]. However, the underlying molecular mechanism involved with the enhancement of GLP‐1 by DHM administration remains to be further elucidated.

Recent studies have found that intestinal regional immunity is closely related to the development of insulin resistance, obesity, and other diseases. Intestinal intraepithelial lymphocytes (IELs) are a unique population of T cells located in the intestinal mucosa [16, 17]. Its different subtypes and functional disorders play an important role in the occurrence of various metabolic diseases [18, 19]. It has been reported that integrin β7^+^ IELs are involved in the development of insulin resistance via regulating the frequency of L cells and level of GLP‐1 [19]. The result suggested that some special subsets of intestinal IELs may affect the expression level of gut‐derived GLP‐1 and regulate the metabolism of the body. Our previous work showed that DHM increased the proportion of ILC3 cells and IL‐22 secreted by ILC3 cells in colonic lamina propria, and promoted the expression level of IL‐22, implying a potential role of DHM on the modulation of intestinal regional immunity [20]. In this study, we hypothesized that DHM could promote GLP‐1 level and improve insulin resistance in HFD‐induced mice by modulation of gut microbiota and the metabolites, which might be associated with modulation of the intestinal L cells and certain subsets of IELs cells.

## 2 Materials and Methods

### 2.1 Animals and Experimental Protocol

Animal experiments were approved by the ethics committee of the Army Medical University (Chongqing, China; Approval AMUWEC20211008) and performed following the guidelines of the center of laboratory animals at the Army Medical University. C57BL/6 mice (specific pathogen free [SPF] grade, male, 6–8 weeks old) were purchased from Hunan SJA Laboratory Animal Co. Ltd. (Hunan, China) and allowed 1 week of acclimatization. The mice were maintained under SPF conditions in a controlled facility with free access to chow and water (temperature 20–22°C, humidity 45% ± 5%, 12 h light/dark cycle).

Mice were randomly divided into three groups with different diets administration for 8 weeks: (1) Control group (*n* = 10): chow diet (10% kcal from fat, 70% kcal from carbohydrate, and 20% kcal from protein); (2) HFD group (*n* = 10): HFD (45% kcal from fat, 35% kcal from carbohydrate, and 20% kcal from protein); (3) HFD with DHM (MUST, China) group (*n* = 10): HFD containing 0.6% DHM (equivalent to 300 mg/kg·B.W./day). Body weight and food intake were measured once a week during the experiments. Average daily food intake (g/day/mouse) was calculated. Feces samples were collected at the 8^th^ week for 16S rRNA, untargeted metabolomics and bile acid (BA) assessment. Mice were fasted overnight and then anesthetized for harvesting blood and DPP‐4 enzyme inhibitor (final concentration of 5 µM) was added. The blood was centrifuged at 3000 × *g* for 10 min for serum isolation. Intestinal tissues were collected immediately after euthanasia, the IELs were isolated by flow cytometry.

For the antibiotic (Abx) treatment experiment, mice were fed with HFD supplemented with or without 0.06% DHM for 8 weeks and were continuously intervened with water containing Abxs for 4 weeks from the 5^th^ to 8^th^ week. Abx intervention was carried out by adding Abx mixture (vancomycin hydrochloride 0.5 g/L, neomycin sulfate 1 g/L, metronidazole 1 g/L, and ampicillin 1 g/L) into sterile water to replace daily drinking water.

For gut microbiota transplantation experiment, mice were administrated with HFD with or without 0.06% DHM for 8 weeks and were fed with Abxs to eliminate gut microbiota at the 5^th^ and 6^th^ week. From the 7^th^ to 8^th^ week, the supernatant of the diluted fecal samples from donors of HFD or HFD + DHM mice was transplanted by the stomach gavage. In detail, fresh fecal samples of mice in HFD group or HFD + DHM group were collected with sterile 1.5 mL EP tubes at the end of the 8^th^ week, and stored in a refrigerator at −80°C for later use (200 mg per animal per time). During gut microbiota transplantation, 200 mg/mouse fecal sample was diluted with 2 mL sterile sodium chloride solution (0.9%), and the fecal resuspension was obtained by vortexing for 5 min. After centrifugation, the precipitate and supernatant were obtained, and the supernatant was retained for gastric perfusion. At the time of intragastric administration, the body weight of the mice was weighed, and intragastric administration was performed according to the body weight of the mice, and the amount of intragastric administration was about 10 µL/g. Mice were gavaged daily during 7^th^–8^th^ week.

### 2.2 Biochemical Analysis

Serum glucose, triacylglycerol (TG), low‐density lipoprotein cholesterol (LDL‐C), high‐density lipoprotein cholesterol (HDL‐C), and cholesterol (CHO) were measured using an automatic biochemical analyzer. Serum GLP‐1 was measured using a commercial biochemical kit (Jingmei, China).

### 2.3 Oral Glucose Tolerance Test (OGTT) and Intraperitoneal Glucose Tolerance Test (IPGTT)

After fasting overnight, the basal blood glucose level was measured first, and mice were given oral glucose or intraperitoneal glucose (2 g/kg), and the blood glucose levels were measured with a blood glucose meter at 0, 15, 30, 60, 90, and 120 min. glucose tolerance impairment was assessed by area under the curve (AUC).

### 2.4 Insulin Resistance Test (ITT)

During the insulin tolerance test (ITT), the mice were intraperitoneally injected with insulin (0.75 U/kg) after fasting for 6 h, and the blood glucose level was measured by blood glucose meter at 15, 30, 60, 90, and 120 min. Insulin resistance was assessed by the AUC.

### 2.5 Immunofluorescence Staining

For immunofluorescence staining of intestine tissues (*n* = 3 per group), the fresh intestinal tissues were embedded in optimal cutting temperature (OCT) compound and cut into frozen intestinal sections. The slides were treated with GLP‐1 antibody and were then exposed to Alexa Fluor 488 goat Antirabbit IgG and Alexa Fluor 594 goat antirabbit IgG. Nuclei were stained with 4,6‐diamidino‐2‐phenylindole‐dihydrochloride (DAPI). After phosphate buffered saline (PBS) washing, slides were mounted using Prolong Gold Antifade Mountant (Life Technologies) and were photographed with a fluorescence microscope camera.

### 2.6 DNA Extraction, 16S rRNA, and Illumina MiSeq Sequencing

Microbial genomic DNA was extracted from each stool sample Using E.Z.N.A. Soil DNA Kit (Omega Bio‐tek, USA). The extracted DNA was detected on a 1% agarose (biowest, ES) gel, and the concentration and purity of DNA were determined using a NanoDrop 2000 ultra‐micro spectrophotometer (Thermo Fisher Scientific, USA). The V3–V4 hypervariable region of the bacterial 16S rRNA gene was amplified using primer pairs. 20 µL PCR reaction system includes 1 µL (10 ng) template DNA, 0.8 µL (5 µM) forward and reverse primers each, 0.4 µL FastPfu DNA Polymerase, 4 µL 5 × FastPfu Buffer, and 2 µL (2.5 mM) dNTPs. The PCR reaction conditions were listed as follows: initial denaturation at 95°C for 30 min, followed by 30 cycles of denaturation at 95°C for 30 s, annealing at 55°C for 30 s, extension at 72°C for 45 s, and stable extension at 72°C for 10 min until that end of the reaction. The PCR products were examined by gel electrophoresis on a 2% agarose gel, and then the PCR products were purified using AxyPrep DNA Gel Extraction Kit (Axygen Biosciences, Axygen, USA) according to the manufacturer's instructions. PCR products were quantified using a Quantus Fluorometer (Promega, USA). According to the sequencing requirements of each sample, the corresponding proportion of mixing was carried out. Use the NEXTFLEX Rapid DNA‐Seq Kit to build the database. The NovaSeq PE250 platform of Illumina was used for sequencing. The sequences were operational taxonomic units (OTU) clustered according to 97% similarity using the UPARSE software (http://drive5.com/uparse/). All data analysis was carried out on the Meiji Biological Cloud Platform (https://cloud.majorbio.com).

### 2.7 Untargeted Metabolomics Analysis

A 50 mg fecal sample was placed in a 2 mL of centrifuge tube with a 6 mm diameter ground bead. Metabolites were extracted with 400 µL of extract (methanol:water = 4:1 V/V) containing 0.02 mg/mL internal standard (L‐2‐chlorophenylalanine). The sample solution was triturated for 6 min (−10°C, 50 Hz) in the cryo‐tissue triturator, and then extracted with low‐temperature ultrasound for 30 min (5°C, 40 kHz). The sample was kept at −20°C for 30 min, centrifuged for 15 min (4°C, 13 000 × *g*), and the supernatant was transferred to a sampling vial with an inner cannula for analysis. Quality control (QC) samples were prepared by mixing the metabolites of all samples in the same volume. During the instrumental analysis, one QC sample was inserted into every four samples to examine the repeatability of the whole analysis process. The instrument platform of this LC‐MS analysis is the UHPLC‐Q Exactive HF‐X system of Thermo Flying Company. After the operation, the LC‐MS raw data was imported into the metabonomics processing software Progenesis QI (Waters Corporation, Milford, USA) for baseline filtering, peak identification, integration, retention time correction, and peak alignment. Finally, a data matrix of retention time, mass‐to‐charge ratio, and peak intensity is obtained. At the same time, the MS and MSMS mass spectra were matched with the public metabolic databases HMDB (http://www.hmdb.ca/) and Metlin (https://metlin.scripps.edu/), as well as the Meiji self‐built database to obtain metabolite information. After searching the database, the matrix data is uploaded to the Meiji Biological Cloud Platform (https://cloud.majorbio.com) for data analysis. To screen for differential metabolites among the indicated three groups, we set a threshold of VIP > 1.0, FC > 1.0, or <1, *p* < 0.05. The metabolic pathway annotation of the differential metabolites was performed through the KEGG database (https://ww.kegg.jp/kegg/pathway.html), and the pathways involved in the differential metabolites were obtained. Pathway enrichment analysis was performed by the Python package of scipy.stats, and the biological pathways most relevant to the experimental treatment were obtained by Fisher's exact test.

### 2.8 Bile Acids (BAs) Targeted Metabolomics Analysis

Precisely pipette 100 µL of sample, add 50 µL of internal standard working solution (200 ng/mL), then add 350 µL of extraction solution (methanol), vortex and mix for 30 s, perform low temperature ultrasound for 30 min (5°C, 40 kHz), stand for 30 min at −20°C, and centrifuge for 15 min at 4°C, 13 000 × *g*. The supernatant was dried with nitrogen, redissolved with 100 µL of 50% acetonitrile water, vortexed and mixed for 30 s, ultrasonically treated at low temperature for 10 min (5°C, 40 kHz), centrifuged at 4°C for 15 min with 13 000 × *g*, and the supernatant was detected by LC‐MS/MS. The samples were analyzed by LC‐MS/MS using an Exion LC AD liquid phase system in combination with a QTRAP 6500+ mass spectrometer (Shanghai Meiji Biomedical Technology Co., Ltd.).

After the operation, the original data of LC‐MS was imported into Sciex quantitative software OS for automatic identification and integration of each ion fragment with default parameters, and manual inspection was assisted to draw a linear regression standard curve with the ratio of the peak area of the analyte to the peak area of the internal standard as the ordinate and the concentration of the analyte as the abscissa. Sample concentration calculation: substitute the ratio of peak area of sample analyte to peak area of internal standard into the linear equation to calculate the concentration result.

### 2.9 IELs Isolation and Flow Cytometry

After mice were sacrificed, the small intestinal tissues in each group were quickly separated, and the mesentery and adipose tissues were removed in ice‐cold PBS. The intestinal tissues were cut open along the longitudinal axis, and the contents were removed and washed twice in ice‐cold PBS. Small intestinal tissue was cut into small pieces and put into 50 mL EP tubes, each tube was added with 20 mL of digestive juice (5% FBS + D‐Hanks + 1 mM DTT + 5 mM EDTA + 10 mM HEPES), and then shaken for 20 min on a constant temperature shaker at 37°C and 210 × *g*. After shaking, swirl the EP tube containing the digestion solution for 20 s, filter and collect the digestion solution into a new 50 mL EP tube using a 70 µm strainer, and add 20 mL to the tube, repeat the above steps, and collect the filtrate. The pooled digest was centrifuged at 2000 × *g* for 5 min, and the supernatant was discarded. The pellet was resuspended in 9 mL of 40% Percoll and transferred to a 15 mL round‐bottomed EP tube. Add 5 mL of 70% Percoll to the bottom of each EP tube through a pipette, centrifuge at 900 × *g* at 20°C, horizontal density gradient for 20 min, and adjust the acceleration and deceleration of the centrifuge to 0. After centrifugation, the round‐bottomed EP tube was carefully collected to the pipette rack. The liquid was divided into three layers, and the middle white layer was the IELs cells. Use a Buss pipette to suck 4 mL of the intermediate tunica albuginea into a 15 mL EP tube with a sharp bottom, and add 8 mL of washing solution (PBS + 5%FBS) for washing, then centrifuge at 2000 × *g* for 5 min, and collect the precipitate as IELs. The gating strategy of FACS is shown in Supporting Information Data S5.

### 2.10 Cell Culture and Enzyme Linked Immunosorbent Assay (ELISA)

The mouse colon cell line STC‐1 (RRID: CVCL_J405) was purchased from the American Type Culture Collection (ATCC). The cells were cultured in DEME growth medium supplemented with 10% fetal bovine serum at 37°C and 5% CO_2_. Cells were plated into a 24‐well plate with an appropriate density and then exposed to vehicle (DMSO, the control, less than 0.1%) with or without 80 µM of chenodeoxycholic acid (CDCA). After incubation, the culture supernatants were collected and the secreted GLP‐1 was detected according to the manufacturers’ instructions by ELISA assay.

### 2.11 RNA Isolation and Quantitative Reverse Transcription PCR

Total RNA was isolated using 1 mL Trizol (Invitrogen). The cDNA templates were obtained from 500 ng of purified RNA using iScript Reverse Transcription Supermix for real time quantitative polymerase chain reaction (RT‐qPCR, Bio‐Rad, CA). 1×SYBR Green Master Mix buffer (Takara, Otsu, Japan) was used for quantitative RT‐PCR, and assays were performed on a Roche lightCycler 480 II PCR machine. Gene specific primers are listed in Table 1. The targeted gene levels were normalized to β‐actin housekeeping gene levels, and the results were analyzed using the 2^−ΔΔCt^ method.

**TABLE 1:** RT‐qPCR primer sequences.

Gene	Primer sequences(5′→3′)
*FXR*	*Left:CCGGCTAAGGAAGTGCAAAG* *Right:AAACTTGGTTGTGGAGGTCA*
*Gcg*	*Left:GCTTATAATGCTGGTGCAAG* *Right:TTCATCTCATCAGGGTCCTC*
*β‐Actin*	*Left:GTGCTATGTTCTAGACTTCG* *Right:ATGCCACAGGATTCCATACC*

### 2.12 Statistical Analysis

Results were presented as mean ± SD. Statistical significance between two different groups was analyzed using the unpaired Student's *t* test and one‐way ANOVA and Kruskal–Wallis H test was used among three‐group. Statistical analyses were calculated using SPSS 19.0 software. Correlations between BAs and microbiome abundances were performed using Spearman's correlation analysis. Differences between experimental groups were considered significant at *p* < 0.05.

## 3 Results

### 3.1 DHM Improves Insulin Resistance by the Enhancement of Incretin Effect in HFD‐Induced Mice

**FIGURE 1:**
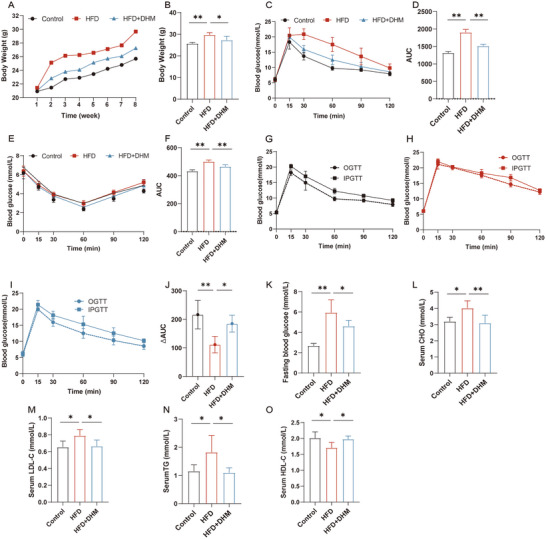
DHM improves insulin resistance by the amelioration of incretin effect in HFD‐induced mice. (A) Body weights at each week among different groups (*n* = 5). (B) Body weight at the end of experiment among different groups (*n* = 5). (C) Oral glucose tolerance test (OGTT) (*n* = 5). (D) Area under the curve (AUC) of OGTT. (E) Insulin resistance test (ITT) (*n* = 5). (F) AUC of ITT. (G) IPGTT and OGTT of Control group (*n* = 5). (H) IPGTT and OGTT of HFD group (*n* = 5). (I) IPGTT and OGTT of HFD + DHM group (*n* = 5). (J) The ΔAUC is indicated by the shaded portions in G–I. (K) Fasting blood glucose at the end of experiment. (L) Serum CHO, (M) LDL‐C, (N) TG, and (O) HDL‐C among different groups (*n* = 6). All data reflect at least two independent experiments, and mean ± SD are plotted. *p < 0.05, **p < 0.01, results as demonstrated by one‐way ANOVA. ANOVA, analysis of variance; CHO, cholesterol; DHM, dihydromyricetin; HDL‐C, high‐density lipoprotein cholesterol; HFD, high‐fat diet; IPGTT, intraperitoneal glucose tolerance test; LDL‐C, low‐density lipoprotein cholesterol; SD, standard deviation; TG, triacylglycerol.

Mice were administrated with HFD with or without DHM for 8 weeks. There was no significant differences in food intake among different groups (Supporting Information Data S1A and B). The body weights were measured each week (Figure 1A), and HFD administration led to a significant increase in the body weight at 8th week, which was ameliorated by DHM intervention (Figure 1B). To further investigate the effect of DHM on glucose homeostasis and insulin sensitivity, the OGTT and ITT assays were conducted. OGTT results showed that DHM could significantly relieve glucose tolerance abnormalities caused by HFD (Figure 1C, D). ITT assay showed that HFD enhanced insulin resistance compared with the Control group, while the changes were alleviated by DHM treatment (Figure 1E, F). To investigate the potential impact of DHM on incretin effect, we performed IPGTT and OGTT experiments. Combined with the results of IPGTT and OGTT, it was showed that HFD could disrupt the incretin effect of mice, which was dominantly restored by DHM administration, as suggested by higher ∆AUC value in the HFD + DHM group; this value represents the area difference between the IPGTT and OGTT curves (Figure 1G–J). Meanwhile, fasting blood glucose level was notably increased in HFD group, which was significantly inhibited by DHM treatment (Figure 1K). In terms of serum lipid metabolism changes, the results showed that compared with the Control group, the serum CHO (Figure 1L), LDL‐C (Figure 1M), and TG (Figure 1N) levels in HFD group were significantly increased, while HDL‐C (Figure 1O) level was significantly decreased (*p* < 0.05). However, the changes of CHO, LDL‐C, TC, and HDL‐C levels in the HFD group were effectively alleviated by DHM treatment, respectively (Figure 1L–O). These results suggested that DHM could effectively improve the HFD‐induced insulin resistance as well as abnormal glucose and lipid metabolism, which might be related to the amelioration of the incretin effect.

### 3.2 DHM Increases Serum GLP‐1 Level by Improving GLP‐1 Secretion in Intestine and Inhibiting GLP‐1 Decomposition in HFD‐Induced Mice

**FIGURE 2:**
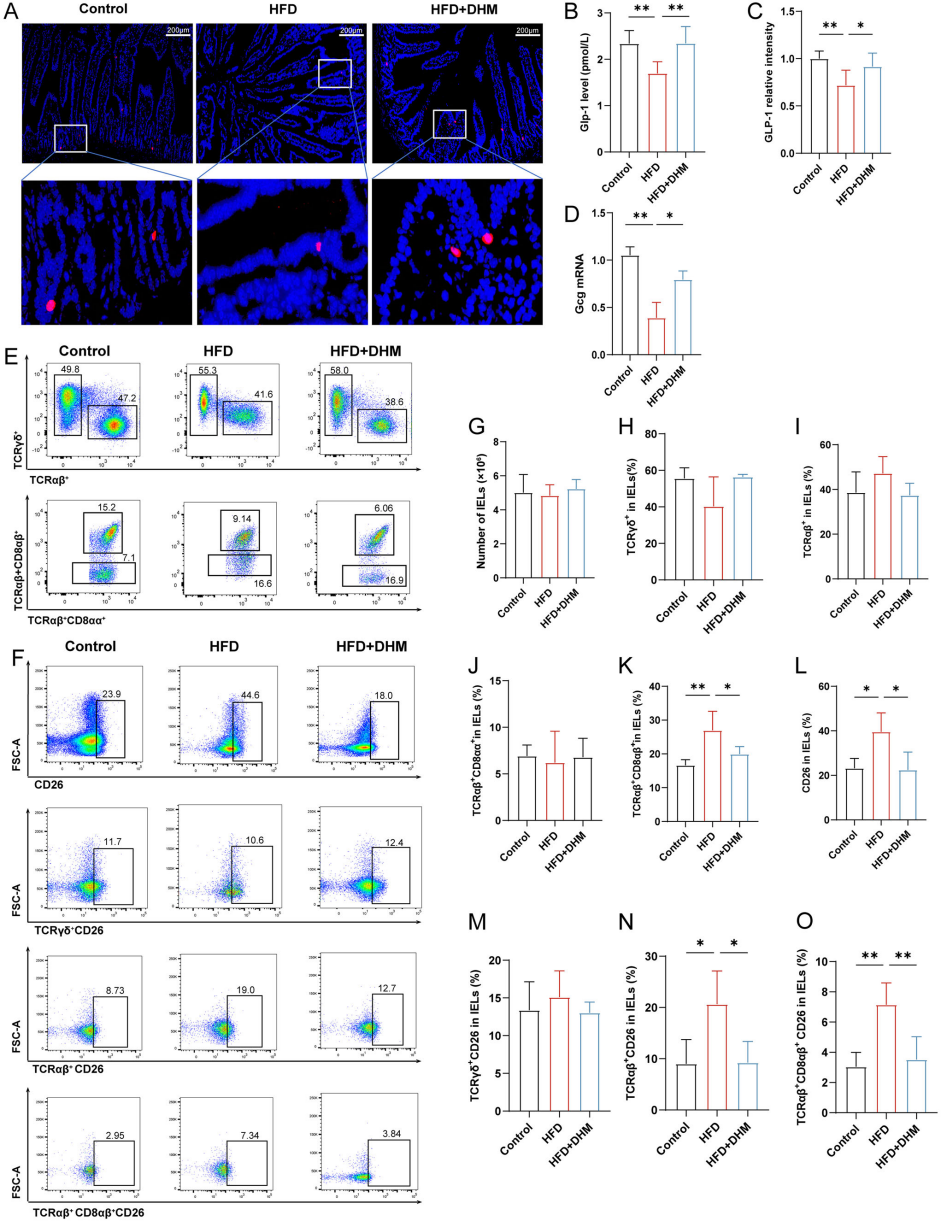
DHM increases serum GLP‐1 level by enhancing GLP‐1 secretion in the intestine and inhibiting GLP‐1 decomposition in HFD‐induced mice. (A) Representative photograph of sections of small intestine stained with anti‐GLP‐1 antibody. Blue fluorescence indicates DAPI and red fluorescence indicates GLP‐1 (*n* = 3 per group). Mice fed with Control, HFD, HFD + DHM over 8 week time. (B) Relative intensity of GLP‐1 among groups (*n* = 6). (C) The serum GLP‐1 level among groups (*n* = 6). (D) RT‐qPCR measurements of the expression levels of Gcg mRNA in small intestine tissues of mice fed with different diets (*n* = 3). (E) Flow fractionation of IELs subsets (*n* = 5). (F) The expression of CD26 in IELs subsets (*n* = 5). (G) Number of IELs in three groups (*n* = 5). (H) The proportion of TCRγδ^+^ IELs in the indicated groups (*n* = 5). (I) The proportion of TCRαβ^+^ IELs in the indicated groups (*n* = 5). (J) The proportion of TCRαβ^+^CD8αα^+^ IELs in the indicated groups (*n* = 5). (K) The proportion of TCRαβ^+^CD8αβ^+^ IELs in the indicated groups (*n* = 5). (L) The expression of CD26 in IELs in the indicated groups (*n* = 5). (M) The expression of CD26 in TCRγδ^+^ IELs in the indicated groups (*n* = 5). (N) The expression of CD26 in TCRαβ^+^ IELs in the indicated groups (*n* = 5). (O) The expression of CD26 in TCRαβ^+^ CD8αβ^+^ IELs in the indicated groups (*n* = 5). All data represent at least two independent experiments, mean ± SD are plotted. *p < 0.05, **p < 0.01 compared between the indicated groups results as demonstrated by one‐way ANOVA. ANOVA, analysis of variance; DAPI, 4,6‐diamidino‐2‐phenylindole‐dihydrochloride; DHM, dihydromyricetin; GLP‐1, glucagon‐like peptide‐1; HFD, high‐fat diet; IEL, intestinal intraepithelial lymphocyte; RT‐qPCR, real‐time quantitative polymerase chain reaction; SD, standard deviation.

We used an immunofluorescence assay to investigate the expression of GLP‐1 protein in the small intestine of mice (Figure 2A). The results showed that GLP‐1 expression was significantly decreased in HFD group compared with the Control group, while the GLP‐1 expression was enhanced after DHM treatment (Figure 2B). Meanwhile, DHM increased serum GLP‐1 level (Figure 2C) and the relative expression of glucagon gene (Gcg) mRNA in intestinal tissue (Figure 2D). Collectively, the results showed DHM enhanced intestinal GLP‐1 secretion and serum GLP‐1 level in HFD‐induced mice.

IELs are a kind of special T cell population in the intestinal mucosal epithelium [21], and their different subsets and phenotypes are closely related to many diseases [22]. Thus, we further investigated whether DHM‐induced change of serum GLP‐1 level was resulted from the increase of its synthesis and secretion or the decrease of IELs’ degradation. We used flow cytometry to detect the expression of CD26 of IELs and the change of the proportion of each subset of IELs. The results are shown in Figure 2E, F and Supporting Information Data S2A. No statistically significant differences of IEL numbers were found among different groups (Figure 2G). There are no significant differences of TCRγδ^+^ and TCRαβ^+^ IELs among groups (Figure 2H, I). For the analysis of TCRαβ^+^ IELs, the results showed that the proportion of TCRαβ^+^ CD8αα^+^ IELs showed no significant changes among the groups, while the proportion of TCRαβ^+^ CD8αβ^+^ IELs changed significantly (Figure 2J, K). It showed that HFD caused an increase in the proportion of TCRαβ^+^ CD8αβ^+^ IELs, which was decreased after DHM intervention. The CD26 was highly expressed in the HFD group, which was suppressed after DHM intervention, suggesting the inhibitory effect on CD26 expression by DHM treatment (Figure 2L). There was no significant difference in the proportion of CD26 expressed by TCRγδ^+^ IELs between HFD and HFD + DHM group (Figure 2M). The proportion of CD26 expressed by TCRαβ^+^ IELs was significantly increased in the HFD group and decreased after DHM intervention (Figure 2N). The proportion of CD26 expressed by TCRαβ^+^ CD8αβ^+^ IELs was increased in HFD and decreased after DHM intervention (Figure 2O), but no significant changes had found in the proportion of CD26 expressed by TCRαβ^+^ CD8αα^+^ IELs among the groups (Supporting Information Data S2B). Overall, these date demonstrated DHM could increase GLP‐1 level in the intestine of HFD‐induced mice and inhibit CD26 expression of IELs, particularly the expression of CD26 in TCRαβ^+^ CD8αβ^+^ IELs, finally increase serum GLP‐1 level.

### 3.3 DHM Ameliorates GLP‐1 Level and Insulin Resistance by Modulation of Gut Microbiota in HFD‐induced Mice

**FIGURE 3:**
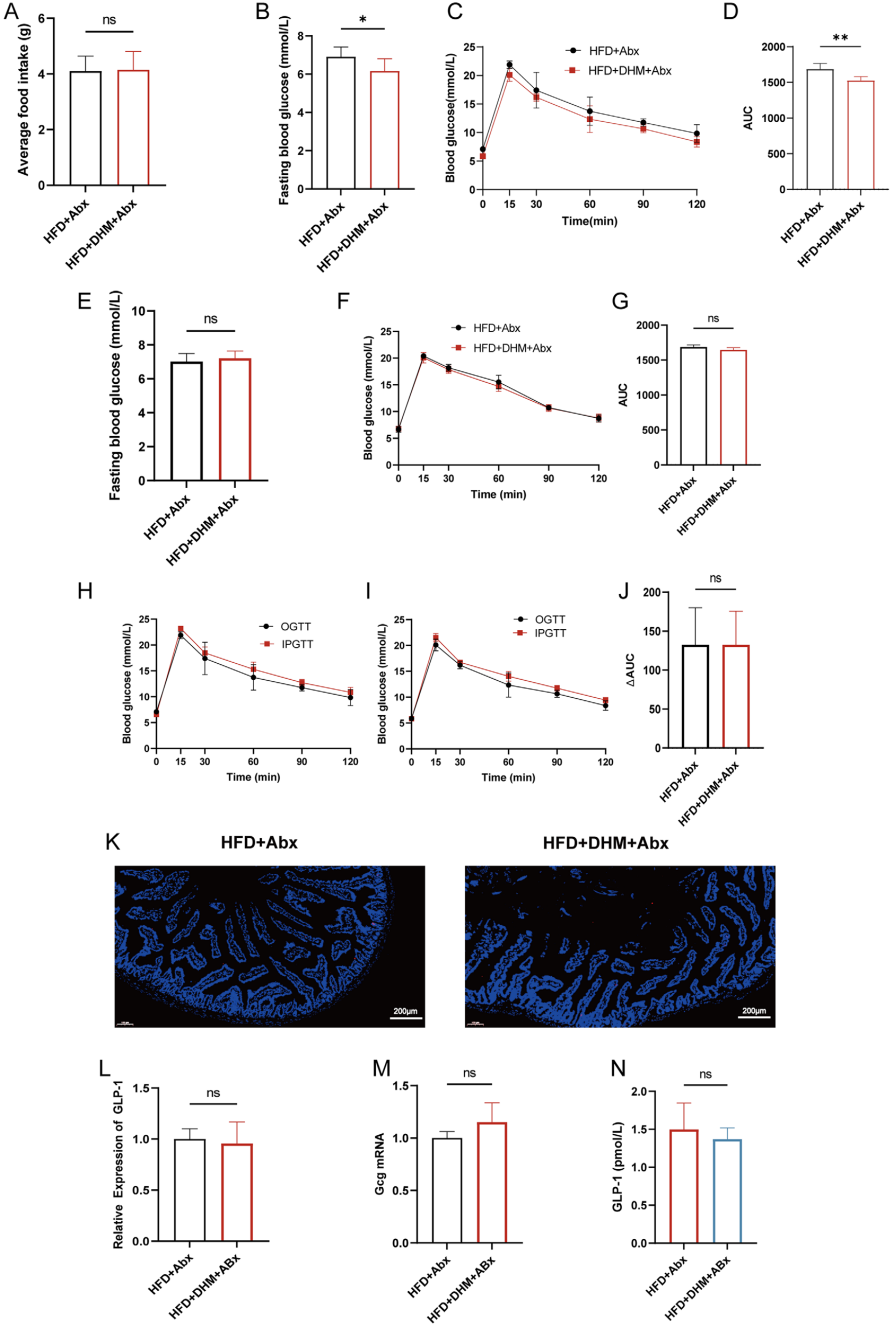
The improvement of DHM on fasting blood glucose, glucose tolerance, incretin and GLP‐1 levels in mice induced by high‐fat diet was significantly weakened after the intervention of antibiotics. (A) Average food intake. (B) Fasting blood glucose at the end of 4 week (*n* = 10). (C) OGTT between HFD + Abx and HFD + DHM + Abx groups at the end of 4 week (*n* = 5). (D) the area under the curve (AUC) in each group before antibiotic treatment at the end of 4 week. (E) Fasting blood glucose after antibiotic treatment at the end of 8 week (*n* = 5). (F) OGTT between two groups after antibiotic treatment at the end of 8 week (*n* = 5). (G) the AUC in each group after antibiotic treatment at the end of 8 week. (H) IPGTT and OGTT of HFD + Abx group (*n* = 5). (I) IPGTT and OGTT of HFD + DHM + Abx group (*n* = 5). (J) The ΔAUC is indicated by the shaded portions in H–I. (K) Representative photograph of sections of small intestine stained with anti‐GLP‐1 antibody. Blue fluorescence indicates DAPI and red fluorescence indicates GLP‐1 (*n* = 3). Mice fed with HFD + Abx and HFD + DHM + Abx over 8 week time. (L) Relative expression of GLP‐1 in two groups (*n* = 3). (M) RT‐qPCR measurements of the expression levels of Gcg in small intestine tissues (*n* = 3). (N) The GLP‐1 level in two groups (*n* = 6). All data reflect at least two independent experiments, and mean ± SD are plotted. *p < 0.05, **p < 0.01, ns, non‐significance compared between the indicated groups results as demonstrated by unpaired Student's *t* test. Abx, antibiotic; DAPI, 4,6‐diamidino‐2‐phenylindole‐dihydrochloride; DHM, dihydromyricetin; HFD, high‐fat diet; GLP‐1, glucagon‐like peptide‐1; IPGTT, intraperitoneal glucose tolerance test; OGTT, oral glucose tolerance test; RT‐qPCR, real‐time quantitative polymerase chain reaction; SD, standard deviation.

In order to verify the role of gut microbiota in the regulation of systemic insulin resistance by DHM, we carried out Abx treatment and gut microbiota transplantation experiments, respectively. Abx treatment experiment results showed there were no significant differences in food intake between the HFD + Abx and HFD + DHM + Abx groups during the Abx treatment experiments (Figure 3A). Meanwhile, we measured the fasting blood glucose at the end of the 4^th^ week and carried out an OGTT experiment. The results showed the impaired glucose tolerance in the HFD + DHM + Abx group was significantly relieved and the fasting blood glucose was significantly decreased, when compared with HFD + Abx group before Abx treatment (Figure 3B–D). The Abx mixture was added to the drinking water of the mice for 4 weeks for Abx treatment. Interestingly, at the end of 8^th^ week, the effect of DHM on fasting blood glucose and glucose tolerance was disappeared (Figure 3E–G). The analysis of IPGTT combined with OGTT showed there was no significant difference between the two groups in incretin effect after Abx treatment (Figure 3H–J). The immunofluorescence assay results showed that there were no significant differences in the GLP‐1 secretion level. Then we examined the expression level of intestinal Gcg mRNA and serum GLP‐1 content between HFD + Abx and HFD + DHM + Abx groups after Abx treatment. The results showed there was no significant difference between the two groups. Overall, gut microbiota plays a key role in the improvement of systemic insulin resistance by DHM (Figure 3K–N).

**FIGURE 4:**
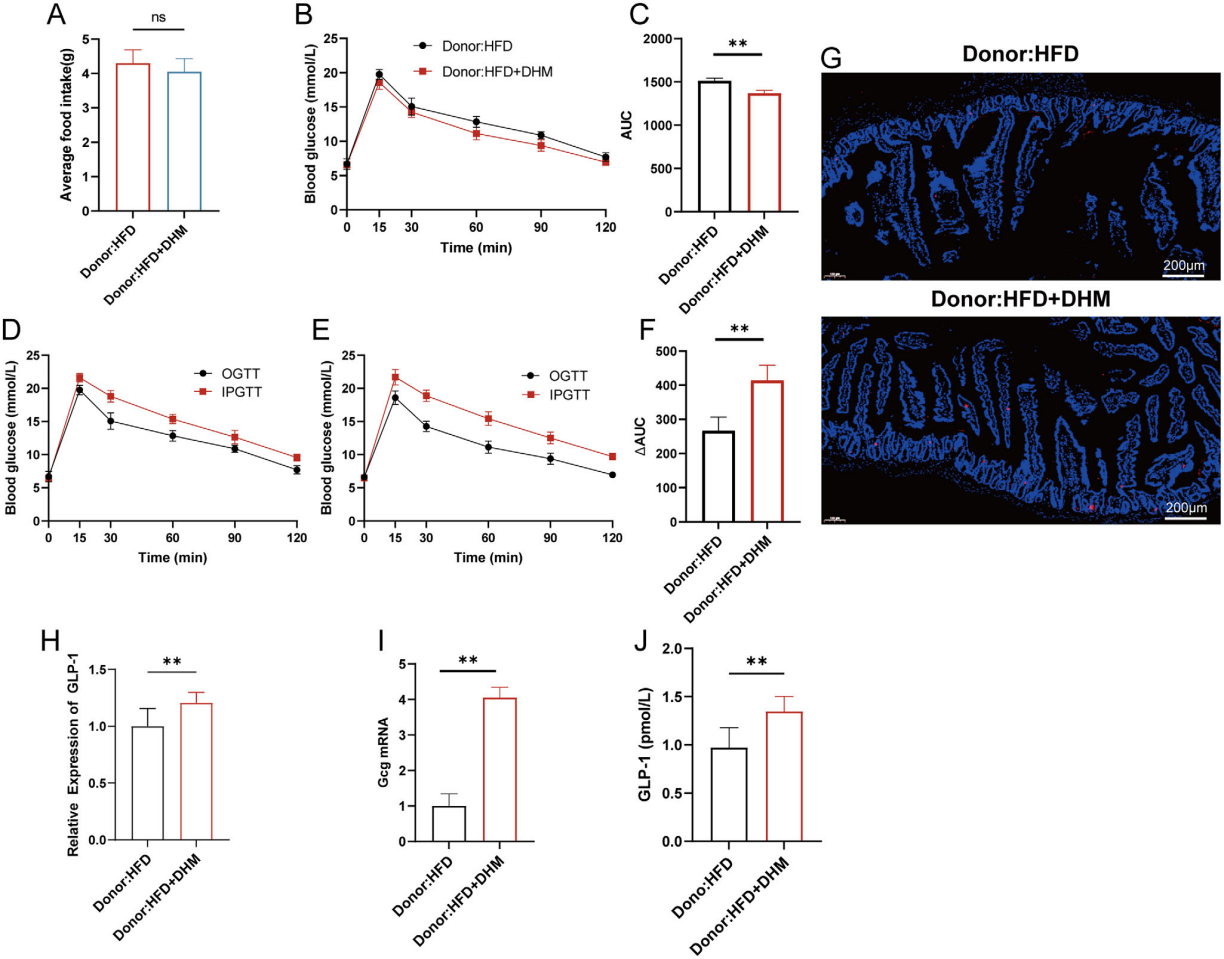
DHM could improve glucose tolerance, incretin, GLP‐1 protein expression in intestinal tissue, and serum GLP‐1 level in mice induced by high‐fat diet after gut microbiota transplantation. (A) Average food intake between Donor:HFD and Donor:HFD + DHM groups during 8 weeks; (B) OGTT between Donor:HFD and Donor:HFD + DHM groups (*n* = 5). (C) The area under the curve (AUC) of OGTT in Donor:HFD and Donor:HFD + DHM groups. (D) IPGTT and OGTT of Donor:HFD group (*n* = 5); (E) IPGTT and OGTT of Donor:HFD + DHM group (*n* = 5); (F) The ΔAUC is indicated by the shaded portions in R–S. (G) Representative photograph of sections of small intestine stained with anti‐GLP‐1 antibody. Blue fluorescence indicates DAPI and red fluorescence indicates GLP‐1 (*n* = 3). Mice fed with Donor:HFD group and Donor:HFD + DHM group over 8 week time. (H) Relative intensity of GLP‐1 in two groups (*n* = 9). (I) RT‐qPCR measurements of the expression levels of Gcg in small intestine tissues (*n* = 3). (J) The GLP‐1 level in two groups (*n* = 6). All data reflect at least two independent experiments, and mean ± SD are plotted. *p < 0.05, **p < 0.01, ns, non‐significance compared between the indicated groups results as demonstrated by unpaired Student's *t* test. DAPI, 4,6‐diamidino‐2‐phenylindole‐dihydrochloride; DHM, dihydromyricetin; HFD, high‐fat diet; GLP‐1, glucagon‐like peptide‐1; IPGTT, intraperitoneal glucose tolerance test; OGTT, oral glucose tolerance test; RT‐qPCR, real‐time quantitative polymerase chain reaction; SD, standard deviation.

Furthermore, we carried out gut microbiota transplantation experiments, in order to verify the role of gut microbiota in the regulation of insulin resistance. We transplanted the gut microbiome of the HFD or HFD + DHM mice into Abx‐treated mice, and fed these recipient mice with HFD or HFD + DHM for 2 weeks to further determine whether the gut microbiota plays a key role in the effects of DHM. The results showed there was no significant difference in food intake between Donor:HFD and Donor:HFD + DHM groups during the experiment (Figure 4A). The OGTT results showed glucose tolerance impairment was relieved after gut microbiota transplantation (Figure 4B, C). The improved effect of DHM on the incretin effect was notably restored, as suggested by the higher ∆AUC value in the Donor:HFD + DHM group; this value represents the area difference between the IPGTT and OGTT curves (Figure 4D–F). The results of immunofluorescence and RT‐qPCR assays showed that the intestinal expression of GLP‐1 and Gcg mRNA, as well as serum GLP‐1 were significantly increased after gut microbiota transplantation (Figure 4G–J). Collectively, these results suggest that the improvement of insulin resistance and GLP‐1 level by DHM treatment may be mediated through the modulation of gut microbiota.

### 3.4 DHM Changes the Gut Microbiota Composition Associated With Insulin Resistance in HFD‐Induced Mice

**FIGURE 5:**
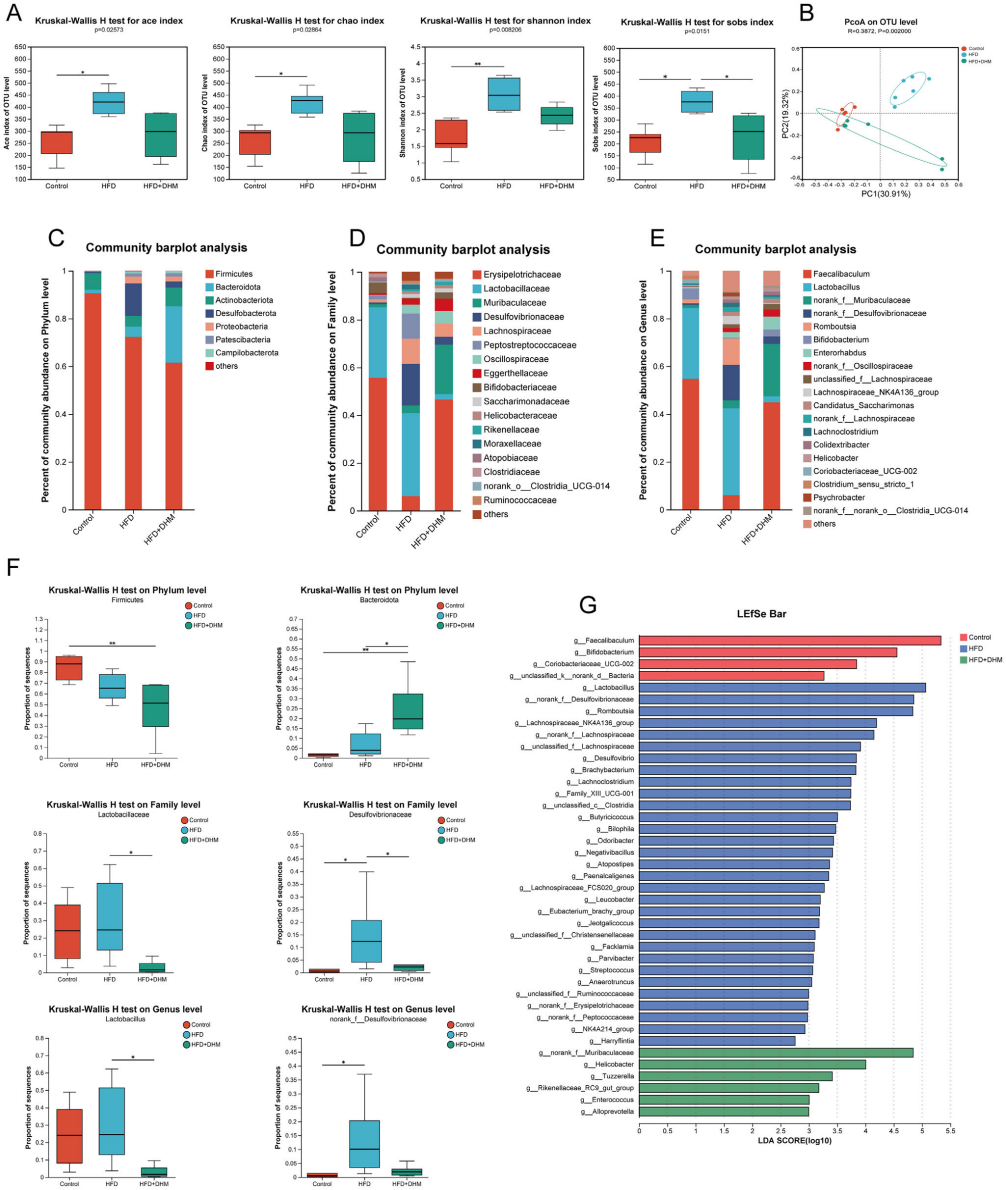
DHM changes the gut microbiota composition associated with insulin resistance in HFD‐induced mice. (A) α‐Diversity analysis. (B) PCoA analysis on OTU level among three groups. (C–E) Bar plot of gut microbiota composition on phylum (C), family (D), and genus level (E). The abscissa/ordinate is the sample name, the ordinate/abscissa is the proportion of the species in the sample, the columns of different colors represent different species, and the length of the column represents the proportion of the species. (F) Relative proportion of sequence in the three groups. (G) Taxa identified by LEfSe of samples from the three groups at the genus level (LDA ≥ 2 and *p* ≤ 0.05). Mean ± SD are plotted, *n* = 6. *p < 0.05, **p < 0.01. Kruskal–Wallis H test was used for the difference analysis of α‐diversity and absolute quantification gut microbiota. DHM, dihydromyricetin; HFD, high‐fat diet; SD, standard deviation.

To investigate how DHM modulates gut microbiota to improve GLP‐1 secretion of intestinal L cells, we evaluated the gut microbiota biospectrum by 16S rRNA sequencing and bioinformatics analysis. The results showed the Ace, Chao, Sobs, and Shannon indexes of the HFD group were significantly different with the Control group (*p* < 0.05). Although Sobs index was different between the HFD and HFD + DHM group, there was no significant difference among the other three indexes (Figure 5A). Meanwhile, the PCoA test on OTU level results showed the gut microbiota diversity distribution in HFD group was significantly different from that in the other two groups (Figure 5B). Collectively, the gut microbiota diversity of mice in HFD group may be significantly changed.

To explore the relationship between DHM and changes of the gut microbiota, we performed a classification analysis of OTUs from primary sequencing data, revealing changes in the microbiota composition of the indicated three groups. The results showed the gut microbiota was changed obviously on phylum, family, and genus levels (Figure 5C–E). On phylum level, the abundance of *Bacteroidata* and *Firmicutes* had no significantly differences between the Control group and HFD group, whereas the abundance of *Bacteroidata* was increased in the HFD + DHM group, compared with the HFD group (Figure 5F). However, DHM significantly decreased the abundance of *Firmicutes* in the HFD + DHM group compared with the Control group. On the family level, compared with the Control group, the abundance of *Desulfovvibrionaceae* in HFD group was increased significantly, which was decreased notably after DHM intervention. The abundance of *Lactobacillaceae* was decreased significantly in HFD + DHM group when compared with the HFD group, and has no significantly differences between the Control group and HFD group (Figure 4F). On the genus level, *norank_f_Desulfovibrionaceae* abundance was increased in the HFD group; the abundance of *Lactobacillus* had a tendency to increase after HFD, but there was no significant difference, and it was decreased significantly after DHM intervention (Figure 5F). The results of LEfEe approach revealed that HFD administration resulted in an increase in abundances of members from the genera *Lactobacillus* and *norank_f_Desulfovibrionaceae*, while *norank_f_Muribaculaceae* were enriched in HFD + DHM group (Figure 5G). Overall, these data suggest that DHM can change the gut microbiota composition associated with insulin resistance in HFD‐induced mice.

In order to analyze the relationship between differential gut microbiota and insulin resistance index, we carried out Spearman correlation analysis. The Spearman correlation analysis showed that the increase of *Desulfobacterota* abundance was positively correlated with glucose, TG, CHO, and body weight, and negatively correlated with GLP‐1. DHM changed the gut microbiota structure of insulin resistance mice induced by HFD (Supporting Information Data S3A–C).

The results of 16S rRNA sequencing of gut microbiota showed that DHM could significantly improve the increase of *Lactobacillaceae* abundance in mice induced by HFD. It has been reported that *Lactobacillaceae* can participate in the regulation of bile acid metabolism by encoding bile salt hydrolase (BSH). The results of genus‐level difference analysis showed that DHM could effectively inhibit the increase of *Lactobacillus* induced by HFD. In order to determine whether DHM has a regulatory effect on the BSH activity of mice induced by HFD, we measured and evaluated the BSH activity of mice in each group (Supporting Information Data S4). The results showed that the BSH activity in HFD group was significantly higher than that of Control group, and the BSH activity in HFD + DHM group was significantly lower than that of the HFD group. This result was similar to the change trend of *Lactobacillaceae* abundance. Therefore, it is speculated that DHM can reshape the gut microbiota structure and regulate BSH activity in HFD‐induced mice.

### 3.5 DHM Could Modulate Intestinal Microbiota Metabolic Pathways in HFD‐Induced Mice

**FIGURE 6:**
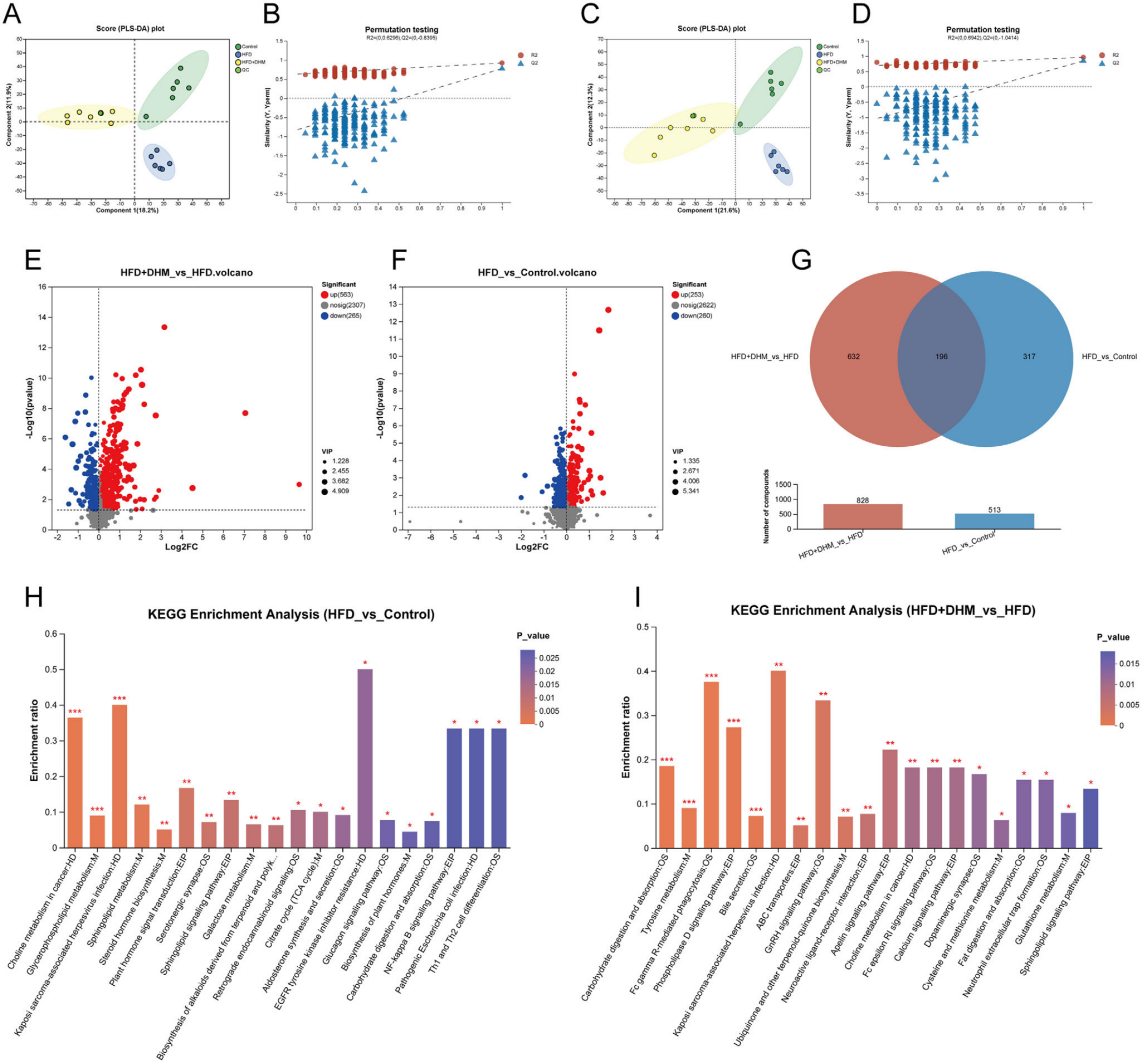
DHM can regulate intestinal metabolism pathways of mice induced by HFD. (A) The positive modes’ PLS‐DA score plots of fecal samples from three groups. (B) Permutation tests of the three groups. (C) The negative modes’ PLS‐DA score plots of fecal samples from three groups. (D) Permutation tests of the three groups. (E, F) Volcano plot of fecal metabolomics of fecal samples showing the significant modes in HFD versus Control (E) and HFD + DHM versus HFD (F). Red dots represent upregulated metabolites and blue dots represent downregulated metabolites. (G) Venn diagram of fecal samples between HFD + DHM versus HFD and HFD versus Control. The orange circles represent the metabolites of HFD + DHM and HFD groups; blue circle represents metabolites that change together in that Control and HFD group; the intersection represents a metabolite of HFD + DHM versus HFD covarying with HFD versus Control. (H, I) KEGG annotation analysis of the changed metabolites based on the differences. (H) KEGG pathway between Control and HFD group. (I) KEGG pathway between HFD and HFD + DHM group. The abscissa represents the pathway name, and the ordinate represents the enrichment rate, which represents the ratio of the metabolite number enriched in the pathway to the metabolite number annotated to the pathway. Indicates a greater degree of enrichment. The column color gradient indicates the significance of enrichment. By default, the darker the color is, the more significant the KEGG term is enriched, ***p < 0.001, **p < 0.01, *p < 0.05, *n* = 6. DHM, dihydromyricetin; HFD, high‐fat die.

In order to verify the effect of DHM on intestinal metabolites in the HFD group, we collected feces from each group at the 8th week, and performed nontargeted metabolomics analysis by LC/MC. The results of PLS‐DA showed that the metabolites of each group were well‐separated from the HFD group, and the results of permutation test showed the prediction ability of the PLS‐DA model was good and there was no over‐fitting phenomenon (Figure 6A–D).

In the comparison between the HFD and the Control groups, 253 differential metabolites were upregulated, while 260 were downregulated (Figure 6E). In the comparison between the HFD + DHM group and the HFD group, 563 differential metabolites were upregulated and 265 downregulated after DHM intervention (Figure 6F). In addition, there were 196 common differential metabolites between the two groups (Figure 6G).

In order to analyze the effects of differential metabolites, we used the KEGG database to conduct pathway enrichment analysis of the measured metabolic sets, and identified the most important biochemical metabolic pathways and signal transduction pathways involved in differential metabolites (Figure 6H, I). There were 43 important differential pathways (*p* < 0.05) between HFD group and the Control group, and 39 differential metabolic pathways (*p* < 0.05) between HFD + DHM group and HFD group. To further analyze the differential pathways, we used the corrected *p* < 0.05 to screen the differential pathways, and the differential metabolite pathways involved were counted (Table 2), between HFD + DHM and HFD group, there were five different pathways, including Fc gamma R‐mediated phagocytosis, phospholipase D (PLD) signaling pathway, tyrosine metabolism pathway, carbohydrate digestion, and absorption pathway and bile secretion pathway. These results suggest that DHM could regulate the intestinal microbiota metabolic pathways of mice induced by HFD, and bile acid metabolism might be highly involved with the improvement of insulin resistance by DHM treatment.

**TABLE 2:** Significant enrichment pathways in HFD versus Control and HFD versus HFD + DHM (*n* = 6 in each group).

Group	Pathways	Corrected *p* value
HFD vs. Control	Glycerophospholipid metabolism	0.0123
	Kaposi sarcoma‐associated herpesvirus infection	0.0437
	Choline metabolism in cancer	0.0003
HFD + DHM vs. HFD	Fc gamma R‐mediated phagocytosis	0.0091
	Phospholipase D signaling pathway	0.0156
	Tyrosine metabolism	0.0091
	Carbohydrate digestion and absorption	0.0057
	Bile secretion	0.0178

### 3.6 DHM Regulates Intestinal Bile Acid Metabolism and Inhibits CDCA and FXR Expression in HFD‐Induced Mice

**FIGURE 7:**
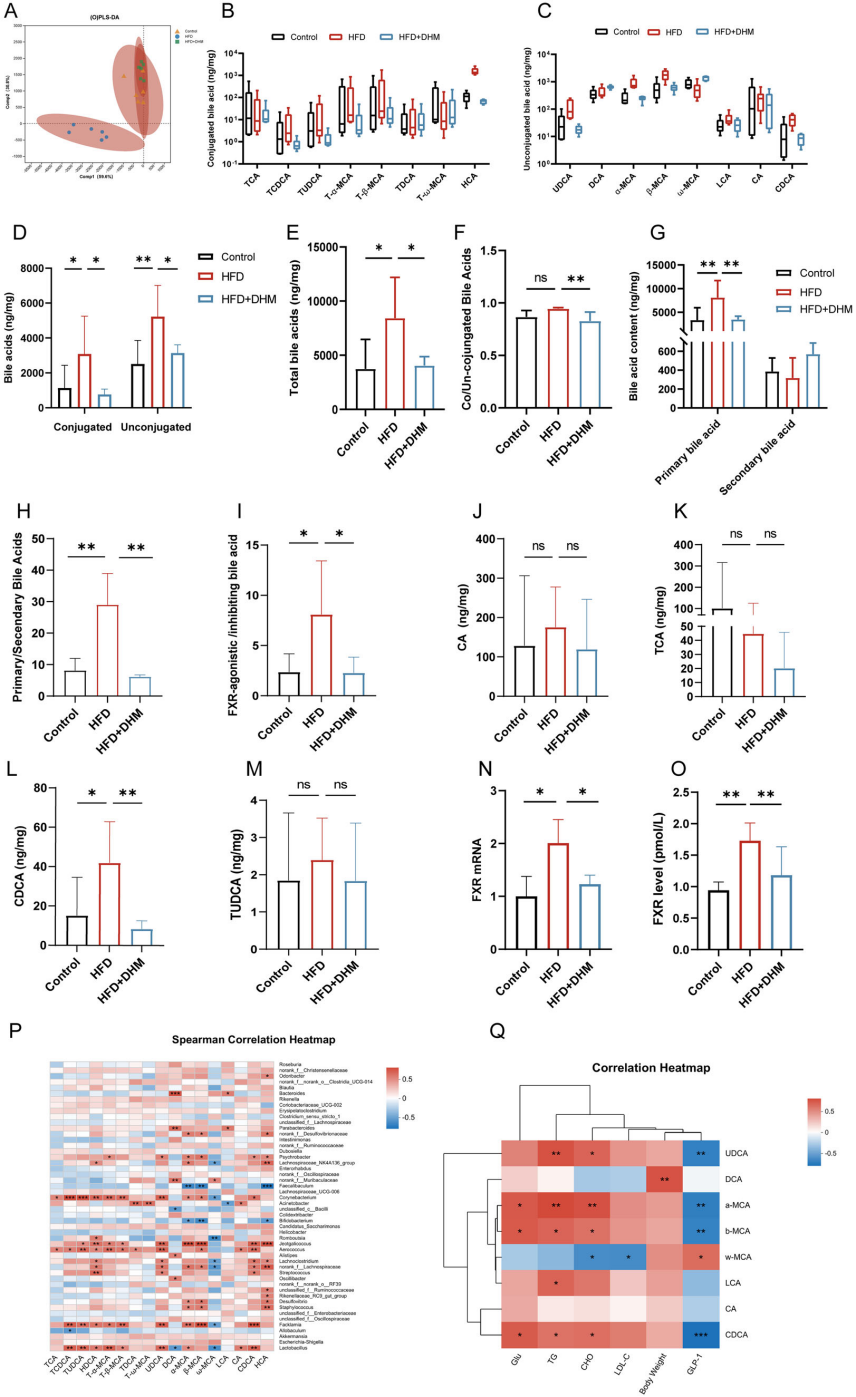
DHM regulates intestinal bile acid metabolism in mice induced by HFD. (A) PLS‐DA score plots of fecal samples from three groups (*n* = 6). (B, C) Statistics of conjugated and unconjugated bile acids in mouse feces (*n* = 6). (D) Differences in conjugated and unconjugated bile acids among the three groups. (E) Difference of total bile acid among groups (*n* = 6). (F) The ratio of conjugated bile acid to unconjugated bile acid in each group (*n* = 6). (G) Contents of primary and secondary bile acids (*n* = 6). (H) The ratio of primary bile acid to secondary bile acid in each group. (I) The ratio of FXR receptor agonistic and inhibitory bile acid abundance (*n* = 6). (J–M) Levels of CA, TCA, CDCA, and TUDCA in mouse fecal samples (*n* = 4–6). (N) Relative Gcg mRNA expression levels in three groups (*n* = 3). (O) The content of FXR in the small intestine of three groups of mice (*n* = 9). (P) Spearman correlation heatmap on genus level between gut microbial and bile acids (*n* = 6). (Q) Spearman correlation heatmap between bile acids and normal indexes (*n* = 6). All data reflect at least two independent experiments, and mean ± SD are plotted. *p < 0.05, **p < 0.01, ***p < 0.0001, ns, non‐significance results as demonstrated by one‐way ANOVA. ANOVA, analysis of variance; CDCA, chenodeoxycholic acid; DHM, dihydromyricetin; FXR, farnesoid X receptor; HFD, high‐fat die; SD, standard deviation; TCA, taurochenodeoxycholic acid; TUDCA, tauroursodeoxycholic acid.

Bile acids exert several functions on regulating intestinal metabolism [23]. To explore the role of bile acid metabolism in the improvement of insulin resistance by DHM, we collected feces and conducted a targeted analysis of bile acid metabolism. PLS‐DA analysis results showed the main components of bile acids in the HFD group were significantly separated from the other three groups, and the main components of HFD + DHM were significantly overlapped with those of the Control group (Figure 7A). The results suggested that the bile acid metabolism in HFD mice was abnormally regulated, and DHM could significantly alleviate the change, which may be consistent with that in the Control group. In order to analyze the changes of intestinal bile acids, we further detected the composition of intestinal bile acids. The conjugated and unconjugated bile acids were counted (Figure 7B, C). The results showed that the level of total bile acids in the HFD group was significantly higher than that of the Control group (Figure 7E). Compared with the Control group, the levels of conjugated bile acids and unconjugated bile acids were significantly increased in the HFD group and decreased after DHM intervention (Figure 7D). However, the results showed there was no significant change in the conjugated/unconjugated bile acid ratio between HFD and the Control groups. DHM significantly reduced the conjugated/unconjugated bile acid ratio between HFD + DHM and HFD groups (Figure 7F). These results suggested that DHM could significantly change the intestinal bile acid metabolism in the HFD group.

Next, we analyzed the changes of primary and secondary bile acids, the results found that HFD significantly increased the content of primary bile acids in the intestine, and DHM significantly decreased the content of primary bile acids, while there was no significant change in secondary bile acids (Figure 7G). The primary/secondary bile acid ratio result showed that DHM could effectively alleviate the increase of primary bile acids (Figure 7H). These data suggest that DHM could correct abnormal primary bile acid metabolism induced by HFD, but had no significant effect on secondary bile acids.

When analyzing the bile acids with metabolic regulation ability, the results showed the ratio of farnesoid X receptor (FXR)‐activated bile acids to FXR‐inhibited bile acids was significantly increased in HFD, compared with the Control group. However, it was significantly decreased after DHM intervention (Figure 7I). FXR agonistic bile acids included cholic acid (CA), taurodeoxycholic acid (TCA), tauro‐β‐muricholic acid (T‐β‐MCA), tauroursodeoxycholic acid (TUDCA), and CDCA. The results showed that CDCA was significantly increased in the HFD group, which was significantly inhibited by DHM treatment. There were no significant changes in other FXR agonistic bile acids among groups (Figure 7J–M). CDCA is reported to be the natural ligand of FXR, so we next tested the FXR expression. The results showed the expression of FXR mRNA and protein were significantly increased in the HFD group and decreased after DHM, respectively (Figure 7N, O).

In order to clarify the relationship between gut microbiota and bile acid metabolic profiles, the correlation between gut microbiota at genus level and bile acid profiles was analyzed. As shown in Figure 7P, the contents of CDCA, UDCA, and TUDCA showed a significant positive correlation with *Lactobacillaceae*. There was a significant positive correlation between *Desulfovibrio* and CDCA, MCA, DCA, and LCA. In order to further expound the relationship between body mass, serum indexes, serum GLP‐1 levels, and bile acid profiles, the correlation analysis was carried out between them and bile acid profile. As shown in Figure 7Q, CDCA was positively correlated with body weight, blood glucose, TG, CHO, and LDL‐C, and negatively correlated with serum GLP‐1 level. There was a significant positive correlation between α‐MCA and TG, CHO, and a significant negative correlation between α‐MCA and GLP‐1. There was a significant positive correlation between β‐MCA and blood glucose and a significant negative correlation between β‐MCA and GLP‐1, respectively.

### 3.7 CDCA Inhibits GLP‐1 Secretion by Stimulating and Increasing FXR Expression in Intestinal L Cells

**FIGURE 8:**
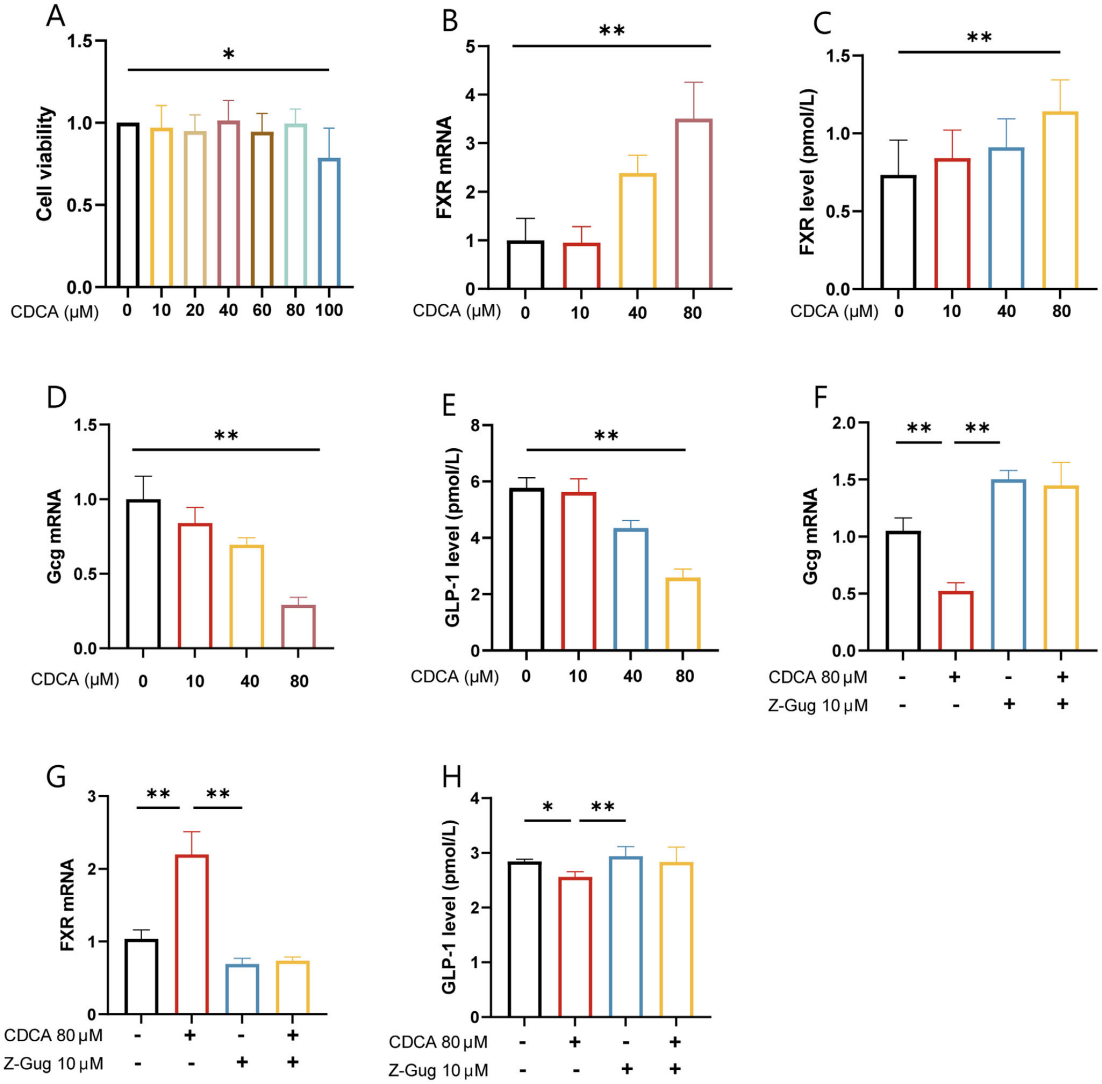
CDCA inhibits GLP‐1 secretion by stimulating and increasing FXR expression in intestinal L cells. (A) Cell viability analysis with 0–100 µM CDCA (*n* = 6). (B) Relative mRNA expression levels of FXR with 0–80 µM CDCA (*n* = 3). (C) FXR level in STC‐1 cell with 0–80 µM CDCA (*n* = 6). (D) Gcg mRNA relative expression levels with 0–80 µM CDCA (*n* = 3). (E) GLP‐1 content secreted by STC‐1 cell with 0–80 µM CDCA (*n* = 6). (F–H) With or without 80 µM CDCA and 10 µM Z‐Gug, STC‐1 secreted GLP‐1 (*n* = 6) and relative expression of Gcg mRNA, FXR (*n* = 3). All data reflect at least two independent experiments, and mean ± SD are plotted. *p < 0.05, **p < 0.01, ns, non‐significance results as demonstrated by one‐way ANOVA. ANOVA, analysis of variance; CDCA, chenodeoxycholic acid; FXR, farnesoid X receptor; Gcg, glucagon gene; GLP‐1, glucagon‐like peptide‐1; SD, standard deviation; Z‐Gug, Z‐guggulsterone.

In order to further verify the effect of CDCA on GLP‐1 secretion by intestinal L cells, we next used STC‐1 cells to investigate how CDCA inhibits GLP‐1 secretion. STC‐1 cells were stimulated with different concentrations of DHM (1, 5, 10, 20, 40, 60, 80, and 100 µM) for 24 h, and then we assessed cell viability by the cell counting kit‐8 (CCK‐8) assay and measuring GLP‐1 levels in the harvested culture supernatant at 0, 10, 40, and 80 µM. Our results showed that CDCA had no significant effect on the proliferation of STC‐1 cells during the range from 1 to 80 µM. At 100 µM, the viability of STC‐1 cells was significantly inhibited (Figure 8A). However, compared with the Control group, 80 µM of CDCA significantly inhibited the GLP‐1 secretion of STC‐1 cells (Figure 8B–D). STC‐1 cells were further treated with 80 µM of CDCA in the presence of absence of FXR antagonist Z‐guggulsterone (Z‐Gug) (10 µM) for 24 h. The results showed that CDCA resulted in an increased expression of FXR mRNA, decreased expression of Gcg mRNA and the secretion of GLP‐1, respectively. In the presence of Z‐Gug, the expression of FXR mRNA was decreased significantly, and the expression of Gcg mRNA and the secretion of GLP‐1 were increased, respectively (Figure 8E–H). To sum up, CDCA promotes the relative mRNA expression of FXR and inhibits the relative mRNA expression of Gcg and the expression of GLP‐1 in intestinal L cells.

## 4 Discussion

**FIGURE 9:**
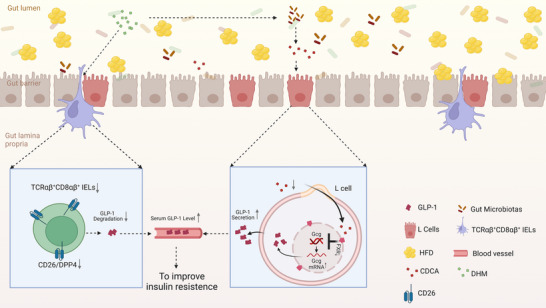
DHM promotes GLP‐1 secretion and improves insulin resistance by modulation of the gut microbiota‐CDCA pathway. DHM could promote the synthesis and secretion of GLP‐1 in intestinal L cells through the “gut microbiota‐CDCA” pathway. Meanwhile, it could regulate the proportion of IELs and the expression of CD26 in IELs and TCRαβ^+^ CD8αβ^+^ IELs, reduce the degradation of GLP‐1, resulting in an increased serum GLP‐1 level and ameliorated insulin resistance. CDCA, chenodeoxycholic acid; DHM, dihydromyricetin; GLP‐1, glucagon‐like peptide‐1; IEL, intestinal intraepithelial lymphocyte.

Insulin resistance has been considered as a common risk factor for a variety of metabolic diseases, which can lead to the occurrence and development of a variety of metabolic diseases [24]. The pathogenesis of insulin resistance is complex. Gut microbiota [25, 26] and metabolite disorder, excessive energy intake, and intestinal immunity [27] play vital roles in the pathogenesis of insulin resistance. At present, there is no effective new drug treatment for insulin resistance [28]. DHM is mostly extracted from a woody vine of the genus *Ampelopsis* of the family Vitaceae and is also extracted from *Hovenia dulcis* Thunb. [29]. DHM has a potential role in the prevention and treatment of a variety of chronic metabolic diseases [30]. A large number of experiments have proved that DHM can reduce atherosclerosis and improve blood glucose and blood lipids [31, 32]. GLP‐1 secreted by intestinal L cells, which can regulate blood glucose and insulin resistance. GLP‐1 is rapidly broken down by DPP‐4/CD26 [6], which is predominantly expressed on the surface of IELs. In this study, we found that DHM could promote the synthesis and secretion of GLP‐1 in intestinal L cells through the “gut microbiota‐CDCA” pathway; meanwhile, it could regulate the proportion of TCRαβ^+^ CD8αβ^+^ IELs and the expression of CD26, reduce the degradation of GLP‐1, thus increasing serum GLP‐1 level and alleviating insulin resistance (Figure 9).

Firstly, we established an HFD‐induced insulin resistance model. Then, we investigated the effects of DHM on glucose metabolism and insulin resistance. The results showed that DHM could significantly reduce the serum glucose, and significantly alleviate insulin resistance and impaired glucose tolerance, which are the core index for evaluating diabetes. In addition, DHM also affected the lipid metabolism of HFD mice. DHM could effectively alleviate the changes of TG, CHO, LDL, and HDL in the HFD group. The incretin effect describes the phenomenon that oral glucose leads to higher insulin secretion than intraperitoneal glucose. The incretin effect refers to the effect of oral glucose that results in higher insulin secretion than intraperitoneal glucose. To determine a potential role in the effects of DHM on the incretin effect, we performed IPGTT and OGTT experiments. The data showed that the incretin effect of HFD mice was significantly weaker than that of the Control mice; DHM could enhance the incretin effect of HFD‐treated mice significantly. By detecting the level of GLP‐1 in the serum, we found that the serum GLP‐1 level in the HFD group was decreased significantly, and increased after DHM intervention. Immunofluorescence results showed the GLP‐1 expression in the intestinal tissue of HFD mice was significantly increased after DHM treatment. The expression of Gcg mRNA was consistent with the result of immunofluorescence. In summary, these results suggest that the beneficial effects of DHM on glucose metabolism and insulin resistance were partially achieved through the enhancement of GLP‐1 level.

DPP‐4/CD26, a serine protease belonging to the Type II transmembrane glycoprotein family, is expressed on the surface of T cells, NK cells, and B cells [33]. It has enzymatic activity and can selectively scavenge N‐terminal dipeptides of polypeptides and proteins containing proline or alanine at the second position. GLP‐1 secreted by intestinal L cells is rapidly degraded by CD26/DPP‐4, limiting its biological function [34]. IELs are a unique T cell population located in the intestinal mucosa, which closely contacts and interacts with intestinal epithelial cells (IECs). It has been found that integrin β7^+^ IELs are involved in the development of insulin resistance. Intestinal L cells in β7^−/−^ mice are significantly increased, GLP‐1 levels are increased, and glucose tolerance is significantly improved. When β7^−/−^ mice are fed with high‐fat and high‐sugar diet, they have significant resistance to obesity, diabetes, and so on [19]. This study suggests that some special subsets of intestinal IELs may affect the expression level of gut‐derived GLP‐1 and regulate the metabolism of the body, so interventions targeting IELs may become a new strategy to regulate the level of GLP‐1. In order to verify whether IELs were involved in the regulation of GLP‐1 physiological concentration maintenance, we separated IELs and detected them by flow cytometry. The results showed that there was no significant change in the number of IELs among groups, but the expression of CD26 in the HFD group was increased and decreased after DHM intervention. The expression of CD26 was decreased after DHM intervention. TCRαβ^+^CD8αβ^+^ IELs were significantly increased in HFD, but the proportion decreased after DHM intervention. CD26 expressed by TCRαβ^+^CD8αβ^+^ IELs was enhanced in the HFD group but was decreased after DHM intervention. Overall, it was suggested that TCRαβ^+^CD8αβ^+^ IELs were involved in the regulation of GLP‐1 degradation.

It has been reported in many research that the gut microbiota plays an important role in the regulation of body metabolism [35, 36]. However, whether gut microbiota plays an important role in the regulation of intestinal GLP‐1 secretion by DHM is unknown. In order to verify whether the gut microbiota plays a vital role in the secretion of GLP‐1, we carried out Abx treatment and transplantation experiments. The experimental results are the same as expected. There were significant glucose intolerance and insulin resistance between HFD + DHM + Abx group and HFD + Abx group before the elimination of gut microbiota. After the elimination of gut microbiota, the difference of insulin resistance between the two groups was inhibited, and GLP‐1 secretion was no different change. In the experiment of gut microbiota transplantation, GLP‐1 secretion inhibition, insulin resistance, and impaired glucose tolerance were observed to be alleviated after gut microbiota transplantation. Our results demonstrated the beneficial effects of DHM on glucose homeostasis and insulin resistance were associated with the gut microbiota.

Gut microbiota, as an important parasitic microorganism in the host intestine, fully participates in the nutritional metabolism of the host and is mainly affected by diet [35, 37]. Obesity induced by HFD has been shown to be associated with gut microbiota dysregulation, as demonstrated by a reduction in gut microbiota diversity and an alteration in gut microbiome composition [38, 39]. Therefore, we measured the gut microbiota to further study its regulatory mechanism. The 16S rRNA analysis results showed that the diversity and abundance of gut microbiota in the HFD group changed significantly. DHM could modulate gut microbiota diversity, especially a decrease of *Desulfovvibrionaceae*, revealing protective features. DHM can effectively regulate the abundance changes of *norank_f_Desulfovibrionaceae* and *Lactobacillus* caused by HFD on the genus level. The regulation of these key microorganisms by DHM is closely related to their therapeutic effects on insulin resistance, which was confirmed by correlation analysis.

Accumulating evidence supports the important role of gut microbiota composition in influencing host metabolism and suggests that targeting gut microbiota composition can regulate host metabolism and reverse insulin resistance [40]. The effect of gut microbiota on host energy metabolism is mainly through amino acids, vitamins, short‐chain fatty acids [41, 42], bile acids [36], and other metabolites, which have changed to varying degrees in obesity. Metabolite profiles of HFD‐induced mice and DHM‐treated mice were analyzed by nontargeted metabonomics. In this study, we show that long‐term HFD interferes with physiological and biochemical processes in mice by affecting clusters of relevant metabolic pathways. Metabolite patterns in HFD‐induced mice were significantly different from those in healthy mice, depending on alterations in glycerophospholipid metabolism, choline metabolism, and Kaposi sarcoma‐associated herpesvirus infection. Meanwhile, *Fc* gamma R‐mediated phagocytosis, PLD signaling pathway, tyrosine metabolism, carbohydrate digestion and absorption, and bile acid metabolism are also closely related to the intervention of DHM on HFD‐induced obesity (Table 2).

Fcγ receptors are a class of cell surface receptors that can bind to the Fc terminus of antibodies and produce intracellular signals mainly through their ITAM activation sequences. Fcγ receptors are involved in many immune system functions, such as phagocytosis, inflammatory mediator release, and antibody‐dependent cellular cytotoxicity. It is an important component of the innate immune response and plays a key role in host defense mechanisms in the uptake and destruction of infectious pathogens.PLD signaling is an important component of the innate immune response. PLD pathway is another important secondary signal transduction system in cells. It has important biological functions, including participating in cell differentiation, proliferation and motility, and enhancing stress response through the endoplasmic reticulum. Tyrosine metabolic pathways describe the many ways in which tyrosine is catabolized or transformed to produce a variety of biologically important molecules. Tyrosine plays a key role in the synthesis of thyroid hormones. Thyroid hormones are produced and released by the thyroid gland and include triiodothyronine (T3) and thyroxine (T4). In this study, the 16S rRNA analysis results showed that the abundance of *Lactobacillus* in the intestinal tract decreased after DHM intervention. Meanwhile, we consulted a large number of research and found that *Lactobacillus* was the main source of BSH. Therefore, we found that BSH content was reduced, after DHM intervention. Overall, the results suggest that DHM may affect bile acid metabolism in mice induced by HFD via gut microbiota.

Bile acids are important physiological agents for the absorption of intestinal nutrients and the secretion of lipids, toxic metabolites, and xenobiotics by the biliary tract [43]. In the intestinal lumen, different bile acids have different physiological functions [44, 45]. Meanwhile, bile acids are also important signaling molecules and metabolic regulators, which can activate nuclear receptors to regulate glucose and energy homeostasis and maintain metabolic homeostasis [46]. So, we performed a targeted metabolic assay for the bile acid metabolism of mice. The results showed that the intestinal bile acid metabolism of HFD mice changed significantly. Total bile acids and primary bile acids were significantly increased. Further analysis of bile acids with biological utility showed that FXR receptor agonistic bile acids increased significantly in the HFD group and decreased significantly after DHM intervention. Subsequently, we analyzed several FXR‐agonistic bile acid concentration changes and found that CDCA was significantly increased in HFD and decreased after DHM intervention. It had been found that the FXR receptor could participate in the regulation of GLP‐1 secretion in intestinal cells. The results of ELISA and RT‐qPCR showed that the expression of FXR was enhanced in the HFD group and decreased after DHM intervention. It has been reported that CDCA is a natural agonist of FXR, which can activate FXR to participate in a variety of metabolic processes in the intestine. Then we detected the expression of FXR mRNA and protein in intestinal tissue by RT‐qPCR and ELISA. The results showed that the expression of FXR mRNA and protein in intestinal tissue in HFD group were increased and decreased after DHM intervention. At the same time, the content of BSH was detected by ELISA. It was found that the content of intestinal BSH in mice was increased by HFD, and the content was decreased by DHM intervention. In the analysis of the correlation between bile acid and gut microbiota, it was observed that the increase of *Lactobacillus* was positively correlated with CDCA. The major source of BSH in the intestine is *Lactobacilli*, which decompose conjugated bile acids into unconjugated bile acids. We hypothesized that the HFD caused an increase in *Lactobacilli* and thus increased BSH content, which in turn increased CDCA synthesis in the intestines.

STC‐1 cells are a commonly used intestinal L cell line in vitro [47].In STC‐1 cells, we found that CDCA could increase the expression of FXR. FXR is a key receptor involved in the secretion of GLP‐1, which can inhibit the transcription of Gcg mRNA and reduce the secretion of GLP‐1. Therefore, we detected Gcg mRNA, and the results showed that the expression of Gcg mRNA decreased. Meanwhile, we treated STC‐1 cells with CDCA and FXR receptor inhibitor Z‐Gug, respectively. The results showed that after the FXR receptor was inhibited, the levels of Gcg mRNA and GLP‐1 were significantly decreased. The results suggest that CDCA inhibits Gcg expression and reduces GLP‐1 secretion by activating FXR.

This study has several limitations. First of all, we have not performed transplantation experiments with specific gut microbiota in mice to verify the role of differential gut microbiota in insulin resistance. Second, intestinal CDCA was found to be changed in the HFD group, but no relevant animal supplementation experiments has been carried out. It is necessary to further improve the research to fully interpret the scientific hypothesis.

## Conflicts of Interest

The authors declare no conflicts of interest.

### Transparent Peer Review

The peer review history for this article is available at https://publons.com/publon/10.1002/mnfr.202400491

## Supporting information



Supporting Information.

Supporting Information.

Supporting Information.

Supporting Information.

Supporting Information.

## Data Availability

The data that support the findings of this study are available from the corresponding author upon reasonable request.
