# Supplementary figures and images for: Dihydromyricetin Promotes Glucagon‐Like Peptide‐1 Secretion and Improves Insulin Resistance by Modulation of the Gut Microbiota‐CDCA Pathway
Source: Mol Nutr Food Res. 2025 Mar 13;69(8):e202400491. doi: 10.1002/mnfr.202400491 (PMC12020986; doi:10.1002/mnfr.202400491)

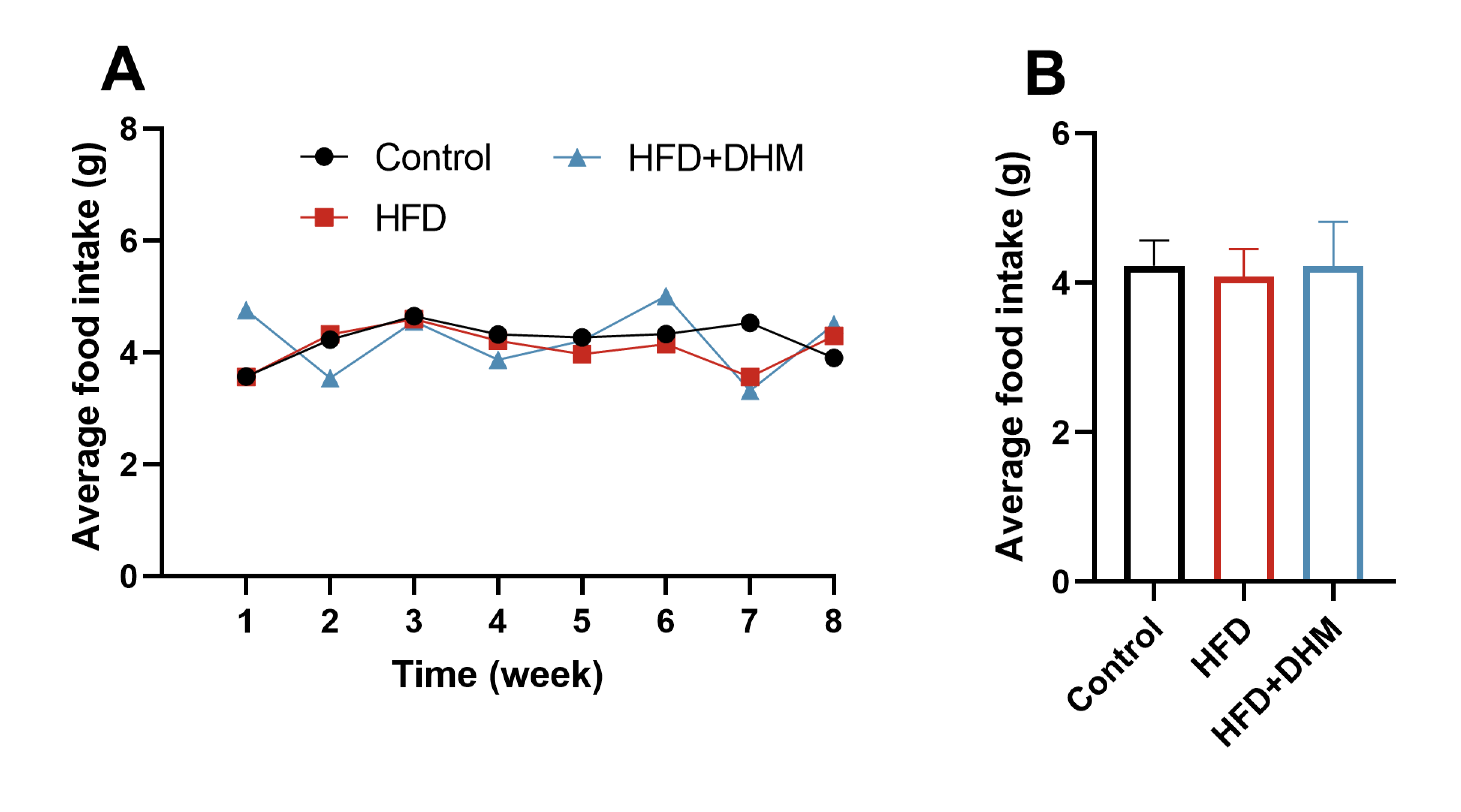

Supplement: Supplementary file 1 — Supporting Information. [file MNFR-69-e202400491-s001.tif]

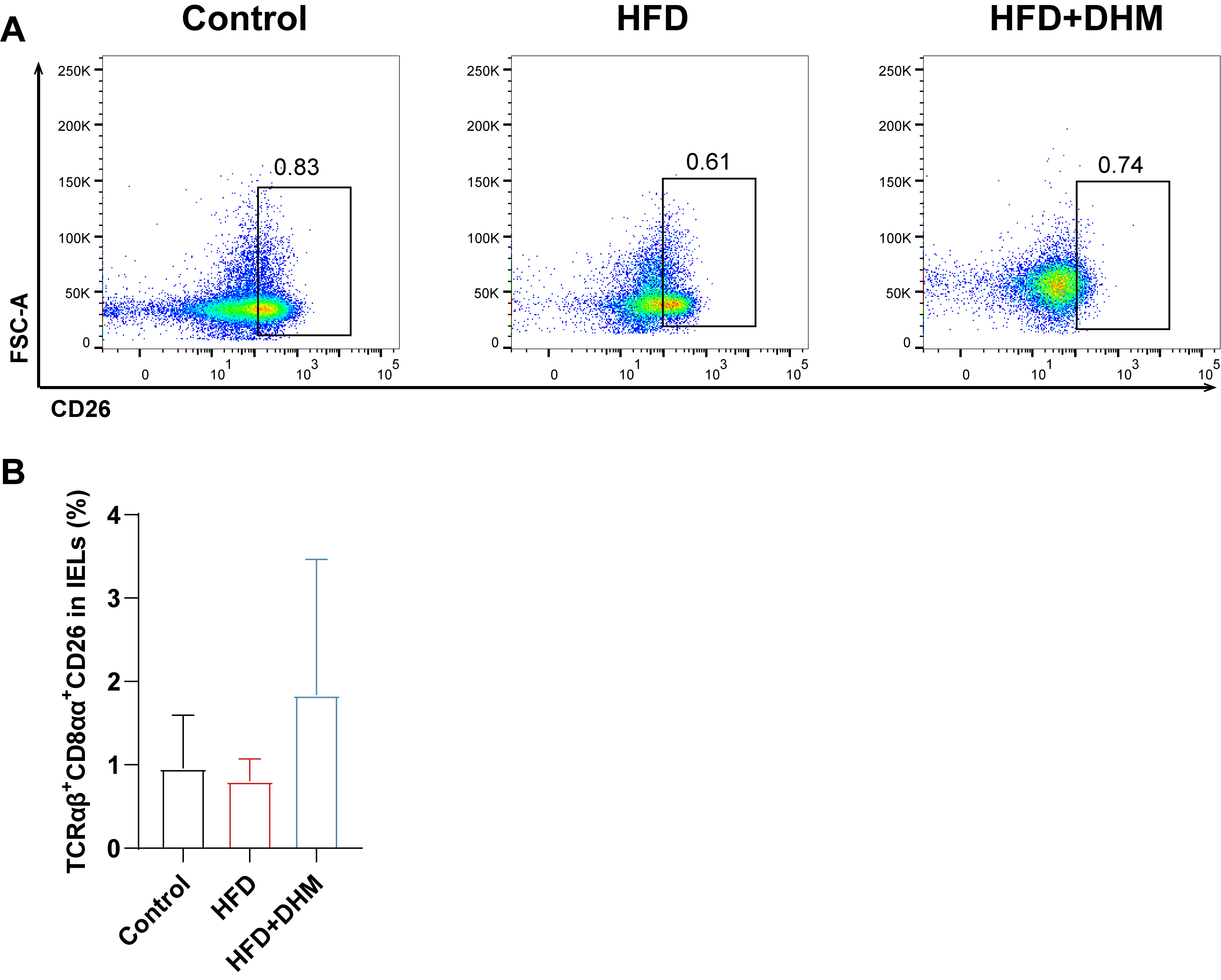

Supplement: Supplementary file 2 — Supporting Information. [file MNFR-69-e202400491-s005.tif]

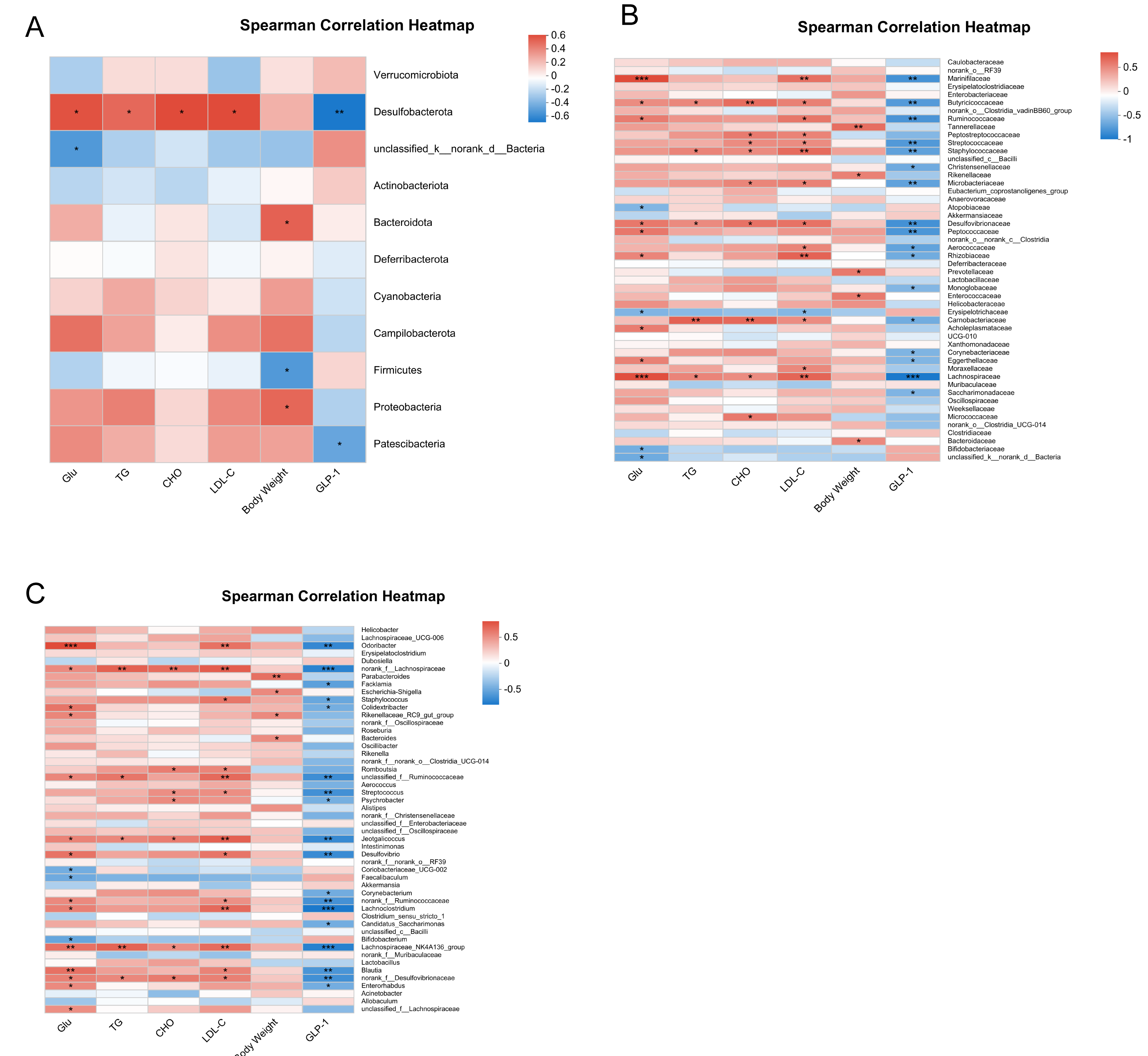

Supplement: Supplementary file 3 — Supporting Information. [file MNFR-69-e202400491-s003.tif]

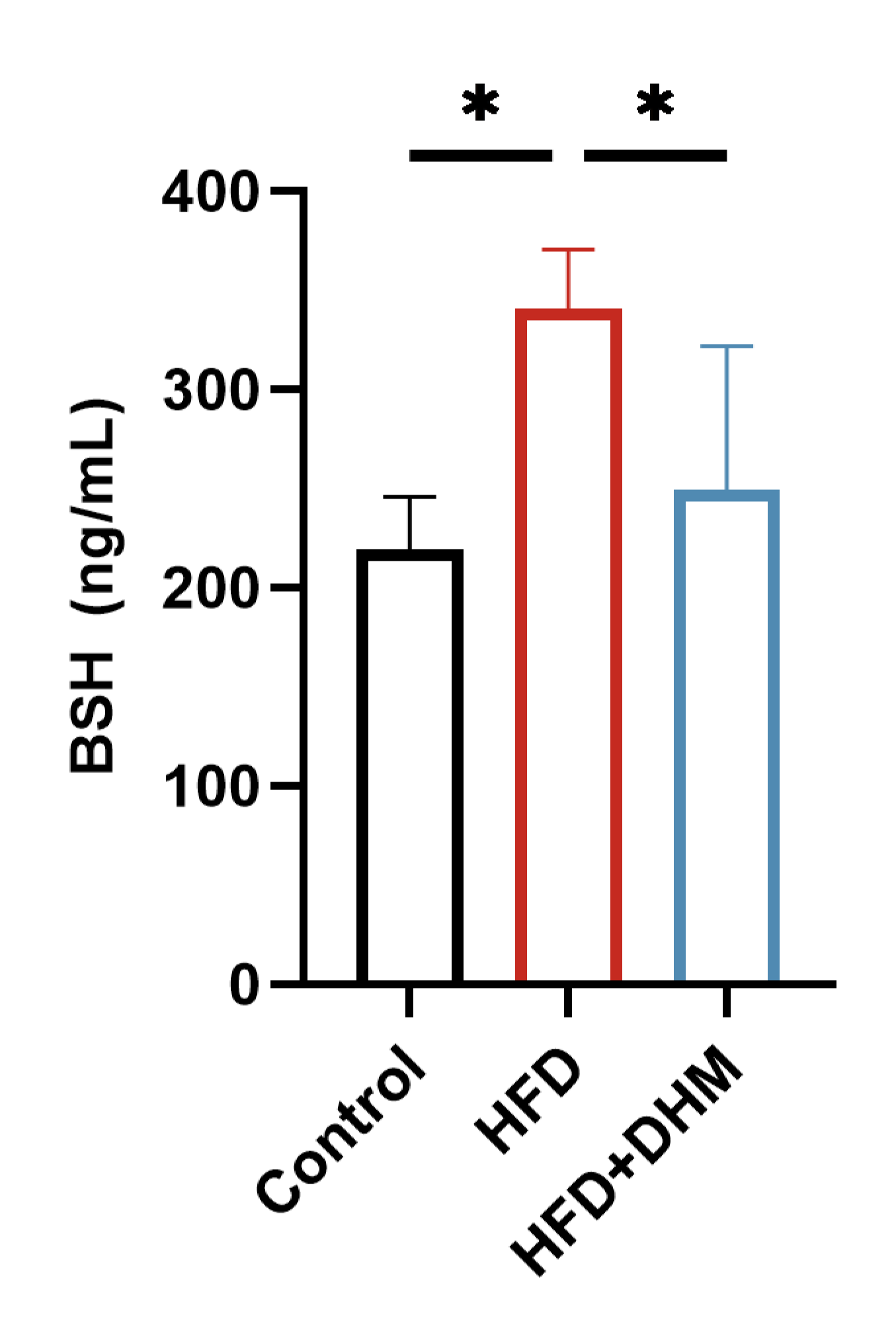

Supplement: Supplementary file 4 — Supporting Information. [file MNFR-69-e202400491-s002.tif]

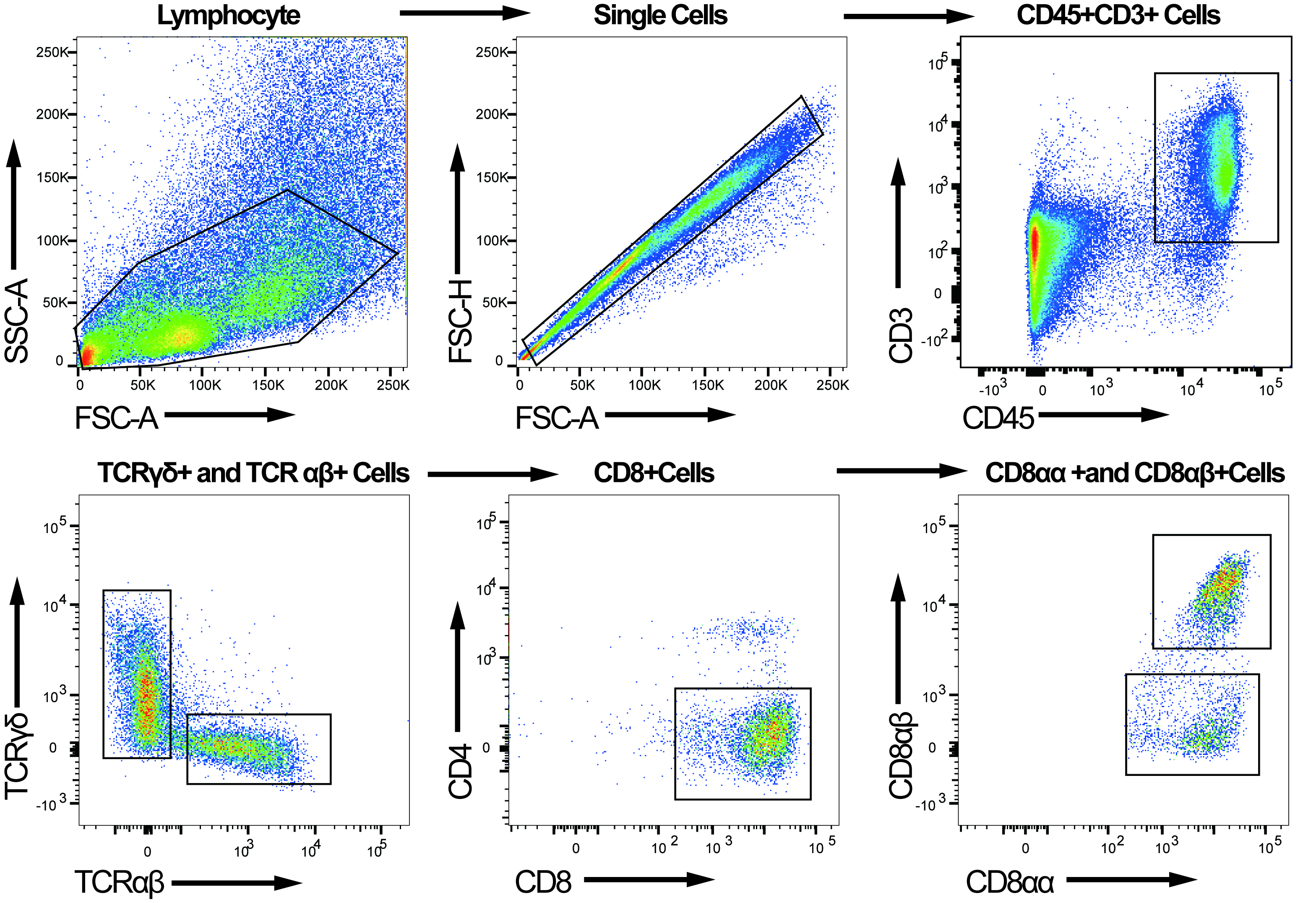

Supplement: Supplementary file 5 — Supporting Information. [file MNFR-69-e202400491-s004.tif]
